# Common DNA sequence variation influences 3-dimensional conformation of the human genome

**DOI:** 10.1186/s13059-019-1855-4

**Published:** 2019-11-28

**Authors:** David U. Gorkin, Yunjiang Qiu, Ming Hu, Kipper Fletez-Brant, Tristin Liu, Anthony D. Schmitt, Amina Noor, Joshua Chiou, Kyle J. Gaulton, Jonathan Sebat, Yun Li, Kasper D. Hansen, Bing Ren

**Affiliations:** 1 Ludwig Institute for Cancer Research, La Jolla, CA USA; 2 Department of Cellular and Molecular Medicine, University of California San Diego, La Jolla, CA USA; 3 Bioinformatics and Systems Biology Graduate Program, University of California San Diego, La Jolla, CA USA; 4 Department of Quantitative Health Sciences, Lerner Research Institute, Cleveland Clinic Foundation, Cleveland, OH USA; 5 McKusick-Nathans Institute of Genetic Medicine, Johns Hopkins School of Medicine, Baltimore, MD USA; 6 Department of Biostatistics, Johns Hopkins Bloomberg School of Public Health, Baltimore, MD USA; 7 Current address: Arima Genomics, San Diego, CA 92121 USA; 8 Department of Pediatrics, University of California San Diego, La Jolla, CA USA; 9 Biomedical Sciences Graduate Program, University of California San Diego, La Jolla, CA USA; 10 Department of Psychiatry, University of California, San Diego, La Jolla, CA 92093 USA; 11 Department of Genetics, Department of Biostatistics, and Department of Computer Science, University of North Carolina, Chapel Hill, Chapel Hill, NC USA; 12 Institute of Genomic Medicine and Moores Cancer Center, University of California San Diego, La Jolla, CA USA

## Abstract

**Background:**

The 3-dimensional (3D) conformation of chromatin inside the nucleus is integral to a variety of nuclear processes including transcriptional regulation, DNA replication, and DNA damage repair. Aberrations in 3D chromatin conformation have been implicated in developmental abnormalities and cancer. Despite the importance of 3D chromatin conformation to cellular function and human health, little is known about how 3D chromatin conformation varies in the human population, or whether DNA sequence variation between individuals influences 3D chromatin conformation.

**Results:**

To address these questions, we perform Hi-C on lymphoblastoid cell lines from 20 individuals. We identify thousands of regions across the genome where 3D chromatin conformation varies between individuals and find that this variation is often accompanied by variation in gene expression, histone modifications, and transcription factor binding. Moreover, we find that DNA sequence variation influences several features of 3D chromatin conformation including loop strength, contact insulation, contact directionality, and density of local cis contacts. We map hundreds of quantitative trait loci associated with 3D chromatin features and find evidence that some of these same variants are associated at modest levels with other molecular phenotypes as well as complex disease risk.

**Conclusion:**

Our results demonstrate that common DNA sequence variants can influence 3D chromatin conformation, pointing to a more pervasive role for 3D chromatin conformation in human phenotypic variation than previously recognized.

## Introduction

Three-dimensional (3D) organization of chromatin is essential for proper regulation of gene expression [1–3] and plays an important role in other nuclear processes including DNA replication [4, 5], X chromosome inactivation [6–9], and DNA repair [10, 11]. Many recent insights about 3D chromatin conformation have been enabled by a suite of technologies based on Chromatin Conformation Capture (3C) [12]. A high-throughput version of 3C called “Hi-C” enables the mapping of 3D chromatin conformation at genome-wide scale [13] and has revealed several key features of 3D chromatin conformation including (1) compartments (often referred to as “A/B compartments”), which refer to the tendency of loci with similar transcriptional activity to physically segregate in 3D space [13–15]; (2) chromatin domains (often referred to as Topologically Associating Domains, or TADs) demarcated by boundaries across which contacts are relatively infrequent [16–18]; (3) chromatin loops, which describe point-to-point interactions that occur more frequently than expected based on the linear distance between interacting loci, and often anchored by convergent CTCF motif pairs [14]; and (4) Frequently Interacting Regions (FIREs), which are regions of increased local interaction frequency enriched for tissue-specific genes and enhancers [19, 20].

Previous studies have used Hi-C to profile 3D chromatin conformation across different cell types [14, 16, 21], different primary tissues [19], different cell states [22], and in response to different genetic and molecular perturbations [23–27], producing a wealth of knowledge about key features of 3D chromatin conformation. However, to our knowledge, no study to date has measured variation in 3D chromatin conformation across more than a handful of unrelated individuals. Several observations demonstrate that at least in some cases DNA sequence variation between individuals can alter 3D chromatin organization with pathological consequences [28]. Pioneering work by Mundlos and colleagues described several cases in which rearrangements of TAD structure lead to gene dysregulation and consequent developmental malformations [29, 30]. In cancer, somatic mutations and aberrant DNA methylation can disrupt TAD boundaries leading to dysregulation of proto-oncogenes [31, 32]. Moreover, many genetic variants associated with human traits by Genome-Wide Association Studies (GWAS) occur in distal regulatory elements that loop to putative target gene promoters in 3D, and in some cases, the strength of these looping interactions has been shown to vary between alleles of the associated SNP [33, 34]. Although these studies demonstrate that both large effects as well as more subtle aberrations of 3D chromatin conformation are potential mechanisms of disease, population-level variation in 3D chromatin conformation more broadly has remained unexplored.

In the present study, we set out to characterize inter-individual variation in 3D chromatin conformation by performing Hi-C on lymphoblastoid cell lines (LCLs) derived from individuals whose genetic variation has been cataloged by the HapMap or 1000 Genomes Consortia [35]. LCLs have been used as a model system to study variation in several other molecular phenotypes including gene expression, histone modifications, transcription factor (TF) binding, and chromatin accessibility [36–42]. These previous efforts provide a rich context to explore variation in 3D chromatin conformation identified in this model system. Through integrative analyses, we found that inter-individual variation in 3D chromatin conformation occurs on many levels including compartments, TAD boundaries, FIREs, and looping interactions. Moreover, we found that variation in 3D chromatin conformation coincides with variation in activity of the underlying genome sequence as evidenced by transcription, histone modifications, and TF binding. Although our sample size is small, we observed reproducible effects of DNA sequence variation on 3D chromatin conformation and identify hundreds of quantitative trait loci (QTLs) associated with features of 3D chromatin conformation. Our results demonstrate that variation in 3D chromatin conformation is readily detectable from Hi-C data, often overlaps with regions of transcriptomic and epigenomic variability, and is influenced in part by genetic variation that may contribute to disease risk.

## Results

### Mapping 3D chromatin conformation across individuals

To generate maps of 3D chromatin conformation suitable for comparison across individuals, we performed “dilution” Hi-C on LCLs derived from 13 Yoruban individuals (including one trio), one Puerto Rican trio, and one Han Chinese trio (19 individuals total; Additional file 2: Table S1). We also included published Hi-C data from one European LCL (GM12878) generated previously by our group using the same protocol [43], for a total of 20 individuals from four different populations. Many of these same LCLs have been used in previous genomic studies [38, 40, 42], allowing us to leverage multiple transcriptomic and epigenomic datasets in our analysis below (Additional file 2: Table S1). Importantly, 18 of these individuals have had their genetic variation cataloged by the 1000 Genomes Consortium [35, 44] (Additional file 2: Table S2), which allowed us to examine the influence of genetic variation on 3D chromatin conformation. Two replicates of Hi-C were performed on each LCL, with each replicate performed on cells grown independently in culture for at least two passages (Additional file 2: Table S3).

**Fig. 1:**
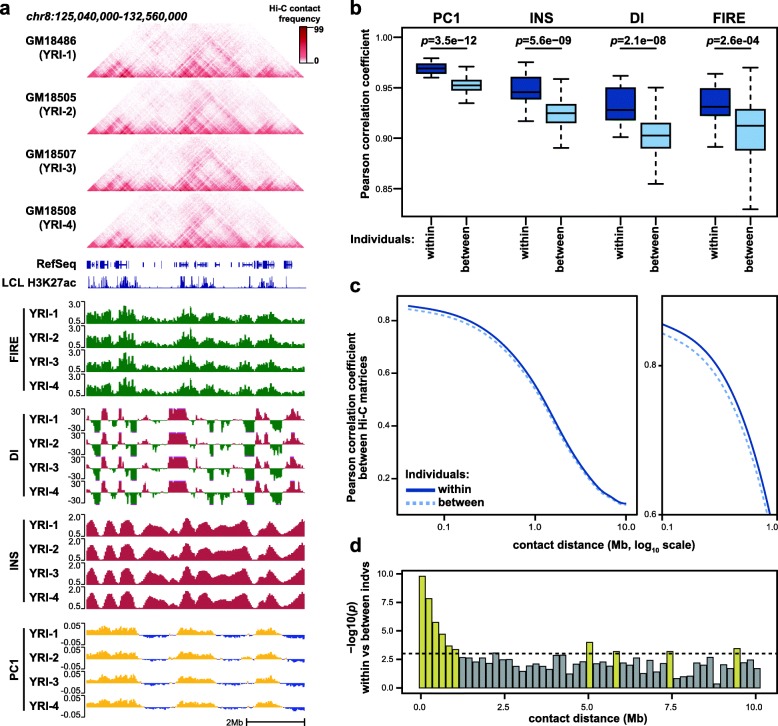
Biological variability in multiple aspects of 3D chromatin conformation. **a** Browser view to illustrate the Hi-C-derived molecular phenotypes examined here: contact matrices, FIRE, DI, INS, and PC1 (chr8:125,040,000-132,560,000; hg19). Only 4 individuals shown here for illustrative purposes. The full set of individuals is shown in Additional file 1: Figure S1. **b** Boxplots show correlation between biological replicates from the same cell line (Individuals = “within”, *N* = 20), and between replicates from different cell lines (Individuals = “between”, *N* = 760). Statistical significance calculated by two-sided Wilcoxon rank-sum test. See Additional file 1: Figure S4a for schematic of shuffling strategy. These and all boxplots in this manuscript show median as a horizontal line, interquartile range (IQR) as a box, and the most extreme value within 1.5*IQR or − 1.5*IQR as whiskers extending above or below the box, respectively. **c** The Pearson correlation coefficient between quantile normalized Hi-C matrix replicates from the same cell line (solid line) or different cell lines (dotted line) is plotted as a function of genomic distance between anchor bins. Distances between 0.1 and 1 Mb are highlighted in the magnified sub-panel to the right. **d** Significance of the difference between the “within” and “between” values in **c** was calculated at multiple points along the distance-correlation curve by two-sided Wilcoxon rank-sum test. Note that the scale of contact distance here is linear. Yellow bars indicate significance exceeding a Bonferroni-corrected significance threshold (dotted line)

All Hi-C data were processed using a uniform pipeline that incorporates the WASP approach [40, 45] to eliminate allelic mapping biases (see the “Alignment with WASP” section). For each sample, we mapped a series of well-established Hi-C-derived features including 40-Kb resolution contact matrices, compartmentalization [13], directionality index (DI) [16], and insulation score (INS) [7] (Fig. 1a; Additional file 1: Figure S1a-c). Compartmentalization is standardly measured by the first principal component (PC1) of Hi-C contact matrices, and thus, we use the acronym “PC1” below to refer to this measure of compartmentalization. DI and INS are both related to TAD boundaries (Additional file 3: Table S4), but they represent different quantitative phenotypes. INS measures the abundance of interactions spanning a given region of interest, whereas DI measures imbalance in the directionality of contacts from a given region of interest (i.e., upstream or downstream). TAD boundaries show a signature of strong insulation reflected by low INS values, as well as an abrupt shift from strong upstream to strong downstream DI. However, strong DI values can occur elsewhere in the genome.

We also identified FIREs and their corresponding “FIRE scores” [19], which measure how frequently a given region interacts with its neighboring regions (15~200 Kb). The concept of FIRE is based on the observation that the frequency of contacts in this distance range is not evenly distributed across the genome, but rather, tends to peak in regions showing epigenomic signatures of transcriptional and regulatory activity (Additional file 1: Figure S1d-e, S2). As we have shown previously [19, 46], FIRE regions often overlap putative enhancer elements (Additional file 1: Figure S1d-e). We did not call “chromatin loops” in this study because our data was not of sufficient resolution, but we used a set of loops previously reported in the LCL GM12878 [14] to examine variation in loop strength among the LCLs in our study. Aggregate analysis shows that these published LCL loops are generally reproduced in our data (Additional file 1: Figure S3).

### 3D chromatin conformation variations between individuals

After uniformly processing all Hi-C data (see the “Hi-C data processing” section), we compared chromatin conformation across LCLs at the level of contact matrices and multiple derived features (PC1, DI, INS, and FIRE). From a genome-wide perspective, each of these 3D chromatin features shows a signature consistent with reproducible inter-individual variation whereby replicates from the same individual (i.e., same LCL) are more highly correlated than datasets from different individuals (PC1 *p =* 3.5e−12, INS *p =* 5.6e−9, DI *p =* 2.1e−8, FIRE *p =* 2.6e−4 by two-sided Wilcoxon rank-sum test; Fig. 1b–d, Additional file 1: Figure S4a-e). The Hi-C data also cluster by population (Additional file 1: Figure S4f-g). We note that this inter-individual and population variation is consistent with an influence from genetic background, but can also be caused by other factors such as sample acquisition [47], batch effects, and other differences between the LCLs.

**Fig. 2:**
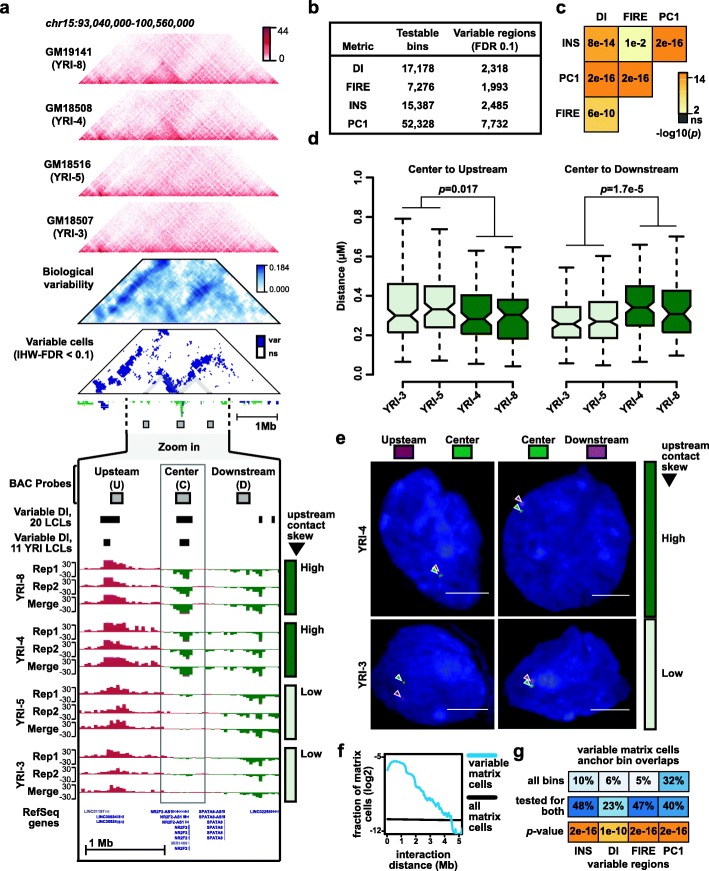
Variable regions of 3D chromatin conformation. **a** Example of a variable region (chr15:93,040,000-100,560,000; hg19). Triangular heatmaps from top to bottom: Four Hi-C contact heatmaps from individuals showing variable 3D chromatin architecture, a heatmap in blue showing the degree of variation measured across LCLs, and a second heatmap in blue showing variable cells in the matrix at IHW-FDR < 0.1 (var = variable, ns = not significant). Standard tracks from top to bottom (zoomed in on chr15:95,482,152-98,025,591; hg19): BAC probes used for FISH experiment in panels **d** and **e**, variable DI regions called using all 20 LCLs, variable DI regions called using just 11 YRI LCLs, 12 DI tracks from four different individuals. For each individual, DI tracks are shown from two biological replicates and from Hi-C data merged across both replicates. As indicated to the right of the tracks, two individuals have strong upstream contact skew in the boxed regions (YRI-4, YRI-8), while the other two individuals have weak or no upstream contact skew in that region (YRI-3, YRI-5). **b** The number of testable bins and significantly variable regions for each 3D chromatin phenotype examined here. **c** Significance of pairwise overlap between different sets of variable regions. *p* values calculated by chi-square test. Additional details in the “Methods” section and Additional file 1: Figure S5 and S6. **d** Boxplots showing the distance between indicated probe sets in four different LCLs. Probe labels same as in panel **a**. *p* values calculated by two-sided Wilcoxon rank-sum test. Number of nuclei measured for each LCL and probe pair, from left to right, are 140, 91, 111, 70, 128, 124, 219, 70. **e** Representative images of nuclei corresponding to panel **d**. **f** Blue line shows the fraction of variable matrix cells distributed across a range of interaction distances. Black shows the fraction of all matrix cells distributed across the same range of interaction distances. **g** Top panel shows the percentage of all variable matrix cell anchor bins that overlap variable INS, FIRE, INS, or PC1 regions, respectively. Middle panel is similar to top, but only including variable matrix cell anchor bins that were also tested for INS, FIRE, INS, or PC1 variability. The shade of blue is scaled with overlap percentage. Bottom panel shows the statistical significance of overlaps as calculated by chi-square test, and plotted with same color scale as (**c**)

Despite generally high correlations of Hi-C data across individuals, we frequently observed regions where 3D chromatin conformation varies reproducibly between individuals (example shown in Fig. 2a, Additional file 1: Figure S5a). To more systematically identify regions of variable 3D chromatin conformation, we used a linear normal model with an empirical Bayes variance estimator (as implemented in the “eBayes” function in *limma* [48]) to identify regions where variation between individuals was significantly higher than variation between two replicates from the same individual. We applied this approach to DI, INS, FIRE, and PC1. For each metric, we first defined a set of testable 40-Kb bins across the genome by filtering out bins with low levels of signal across all individuals or near structural variants that can appear as aberrations in Hi-C maps [49] (see the “*F* test for variable bins” section). We then applied a false discovery rate (FDR) threshold of 0.1 and merged neighboring variable bins, resulting in the identification of 2318 variable DI regions, 2485 variable INS regions, 1996 variable FIRE regions, and 7732 variable PC1 regions (Fig. 2b, Additional file 1: Figure S5b, Additional file 4: Table S5). We note that there is strong overlap between the variable DI, INS, FIRE, and PC1 regions detected across all 20 LCLs and those detected using only the 11 unrelated YRI LCLs, which suggests that potential confounding effects of variation between different populations are not driving the identification of these variable regions (Additional file 1: Figure S5c). Although each metric has a unique set of testable bins, we found significant enrichment for bins that are variable in more than one metric (Fig. 2c, Additional file 1: Figure S5d), indicating that the same regions often vary across multiple features of 3D chromatin conformation.

We next used fluorescent in situ hybridization (FISH) to examine whether variable regions detected by Hi-C are consistent with distance measurements from imaging data. Focusing on a variable DI region on chromosome 15 (Fig. 2a, Additional file 1: Figure S6a-b), we performed FISH in LCLs from four individuals that showed different levels of DI at the variable region being evaluated (YRI-4 and YRI-8 showing high upstream contact bias; YRI-3 and YRI-5 showing no upstream contact bias). We used three BAC probes that hybridize respectively to the variable DI region (“center”, probe covers chr15:96715965-96898793), a region approximately 668 Kb upstream (“upstream”, probe covers chr15:95897555-96047720), or a region approximately 590 Kb downstream (“downstream”, probe covers chr15:97488414-97648104). We found that distances between the center probe and flanking probes vary significantly between individuals with strong upstream contact bias as measured by DI and individuals without this upstream contact bias (Fig. 2d, e, center-upstream distance *p =* 0.017, center-downstream distance *p =* 1.7e−5 by two-sided Wilcoxon rank-sum test). In the two individuals with strong upstream DI signal at the central variable DI region, we found that the center probe is closer to the upstream than the downstream probe, as expected (*p =* 3.2e−3 for YRI-3, *p =* 1.5e−4 for YRI-5 by one-sided Wilcoxon rank-sum test). However, in individuals without upstream DI signal, the center probe is closer to the downstream probe (*p =* 0.021 for YRI-4, *p =* 0.1 for YRI-8 by one-sided Wilcoxon rank-sum test) (Additional file 1: Figure S6c).

**Fig. 3:**
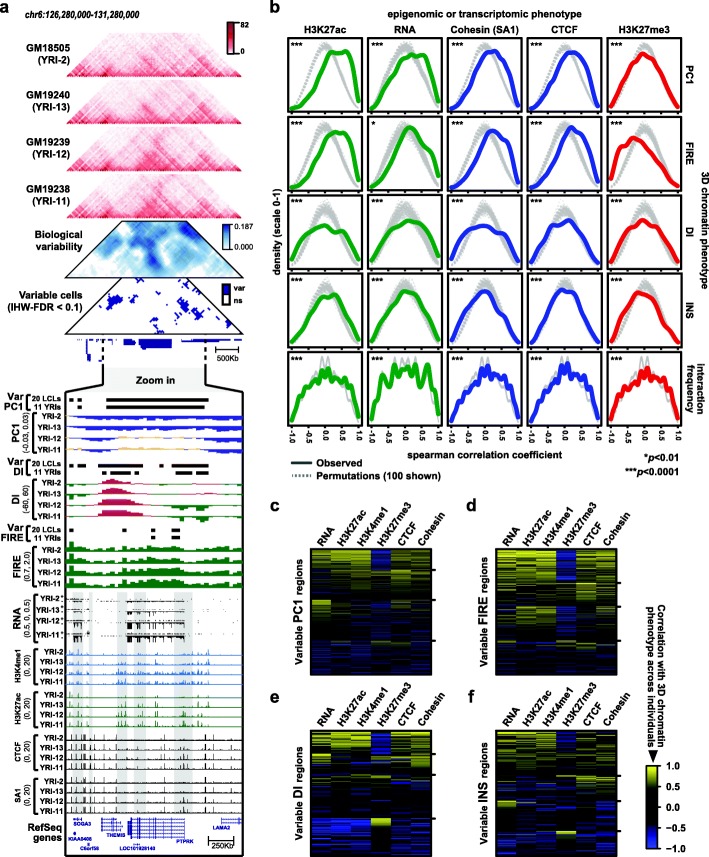
Coordinated variation of the 3D genome, epigenome, and transcriptome. **a** Example of a variable region where 3D chromatin phenotypes are correlated with epigenomic and transcriptomic phenotypes (chr6:126,280,000-131,280,000; hg19). Six triangular heatmaps from top to bottom: Hi-C contact heatmaps from four individuals, variability matrix, and variable cells in the matrix (var = variable, ns = not significant). Standard tracks below show 3D chromatin, epigenomic, and transcriptomic properties from four individuals in zoomed region (chr6:127,680,918-129,416,097; hg19). All ChIP-seq and RNA-seq data in the Figure from Kasowski et al. [42]. **b** Density plots show the distribution of Spearman correlation coefficients at variable regions between the epigenomic or transcriptomic phenotype indicated in the top margin and the 3D chromatin phenotype indicated in the right margin of panel. Gray lines show the distributions from 100 random permutations selected at random from the 10,000 permutations performed (due to plotting limitations). ***p < 0.0001 by permutation test as described in the “Identifying biological variability in Hi-C contact matrices” section, which applies to all observations in this panel except RNA-seq at INS regions (*p =* 0.0018) and RNA-seq at FIRE regions (*p =* 0.0096). **c** Heatmap showing Spearman correlation coefficients between PC1 and multiple epigenomic/transcriptomic phenotypes, arranged by *k*-means clustering (*k* = 4). Tick marks to the right show boundaries between clusters. Each row (*N* = 518) is one variable PC1 region, limited to the subset of variable PC1 regions that contain RNA-seq signal and at least one peak in at least one individual for each ChIP-seq target included here (H3K27ac, H3K4me1, H3K27me3, CTCF, Cohesin). **d** Similar to **c**, showing correlations with FIRE at *N* = 132 variable FIRE regions. **e** Similar to **c**, showing correlations with DI *N* = 265 variable DI regions. **f** Similar to **c**, showing correlations with INS at *N* = 154 variable INS regions

We also sought to identify variable entries in the Hi-C contact matrix itself (“Hi-C contact matrix cells”). To facilitate this search, we used a method called Bandwise Normalization and Batch Correction (BNBC) that we recently developed to normalize Hi-C data across individuals (Fletez-Brant et al. Pre-print: 10.1101/214361). BNBC takes contact distance into account as a covariate because batch effects in Hi-C data can be distance-dependent. To identify variable matrix cells, we performed a variance decomposition on Hi-C contact matrix cells, resulting in a measure of biological variability for each bin in the contact matrix (see example in Fig. 2a and Additional file 1: Figure S5a). To identify matrix cells with significant levels of biological variability, we estimated FDR using the IHW framework [50] to include the distance between anchor bins as an informative covariate. At an FDR threshold of 0.1, we identified 115,817 matrix cells showing significant variability between samples (Additional file 5: Table S6). These variable bins are skewed toward shorter contact distances (Fig. 2f, Additional file 1: Figure S6d-e), likely due in part to higher read counts and thus increased power to detect biological variability at these distances. We observed that the anchor regions of variable matrix cells overlap with variable regions of DI, INS, FIRE, and PC1 more often than would be expected by chance (Fig. 2g; Additional file 1: Figure S6f). We also found that variable matrix cells tend to occur in groups (Figs. 2a and 3a; Additional file 1: Figure S6 g), suggesting that variation in 3D chromatin conformation often affects more than one adjacent genomic window. We did not find evidence that variable matrix cells are skewed toward inter-TAD or inter-compartment interactions (Additional file 1: Figure S6 h-i).

### Coordinated variation of the 3D genome, epigenome, and transcriptome

To investigate the relationship between variation in 3D chromatin conformation and gene regulation, we analyzed multiple published datasets including RNA-seq, ChIP-seq, and DNase-seq data generated from some of the same LCLs in our study (Additional file 2: Table S2). Strikingly, for all external datasets examined here, we see an enrichment for regions where 3D chromatin conformation across individuals is correlated with measures of genome activity in the same 40-Kb bin (see example in Fig. 3a and Additional file 1: Figure S7a). To assign a level of statistical significance to these observations, we approximated the null distribution by randomly permuting the sample labels of external datasets, thus disrupting the link between Hi-C and ChIP/RNA/DNase-seq data from the same individual, but not changing the underlying data structure (see schematic in Additional file 1: Figure S7b). We used these permutations to calculate the bootstrap *p* values in Fig. 3b and Additional file 1: Figure S8.

Among variable PC1 regions, we observed a significant enrichment for regions at which PC1 values across individuals are positively correlated with histone modifications indicative of transcriptional activity including H3K27ac, H3K4me1, and H3K4me3 (Bootstrap *p <* 0.0001; Fig. 3b). The correlations between PC1 and marks of transcriptional activity occur in the expected direction, i.e., higher PC1 values are associated with higher gene expression and more active histone modifications. Similar correlations were apparent in two distinct sets of ChIP-seq data generated by different groups [40, 42], and observed whether we use variable regions identified across all 20 LCLs or only across the 11 unrelated YRI LCLs (Additional file 1: Figure S8). At variable FIRE regions, we similarly found an abundance of regions where FIRE score is positively correlated with marks of cis-regulatory activity including H3K27ac and H3K4me1 (Bootstrap *p <* 0.0001; Fig. 3b, Additional file 1: Figure S8), consistent with the previously reported relationship between FIREs and cis-regulatory activity [19, 46]. At variable DI and INS regions, these metrics tend to be correlated with histone modification levels as well as CTCF and Cohesin subunit SA1 binding (Bootstrap *p <* 0.0001; Fig. 3b, Additional file 1: Figure S8), which are known to influence these 3D chromatin features [16, 51, 52]. For INS, the relationship is directional as expected such that higher CTCF/Cohesin binding corresponds to more contact insulation (i.e., lower INS score). However, at variable DI regions, the correlations are not as clearly directional, consistent with current understanding that the direction of DI (i.e., upstream vs downstream contact bias) is arbitrary relative to strength of CTCF/Cohesin binding. We performed similar analysis on variable cells in the contact matrix and found that the interaction frequency in these matrix cells tends to be correlated with epigenetic or transcriptional properties of one or both corresponding “anchor” bins (Bootstrap *p <* 0.0001; Fig. 3b, Additional file 1: Figure S8). Importantly, for all types of variable regions examined here, we found correlation with RNA-seq signal across individuals, indicating that at least in some regions, variation in 3D chromatin features accompanies variation in gene expression.

We examined further whether 3D chromatin conformation at a given variable region tends to be correlated with a single epigenomic property, or with several properties simultaneously. We found that PC1, FIRE, INS, and DI values across individuals are often correlated with multiple features of active regions (e.g., H3K27ac, H3K4me1, RNA), and anti-correlated with the repressive H3K27me3 histone modification (Fig. 3c, d). For DI and INS, where numerical direction of the score is not as clearly linked to magnitude of gene regulatory activity, we note a larger set of regions with anti-correlation to features of active regions (e.g., H3K27ac, H3K4me1, RNA) and positive correlation with H3K27me3 (Fig. 3e, f). These results demonstrate that variation in 3D chromatin conformation is often accompanied by variation in transcriptional and regulatory activity of the same region.

### Genetic loci influencing 3D chromatin conformation

**Fig. 4:**
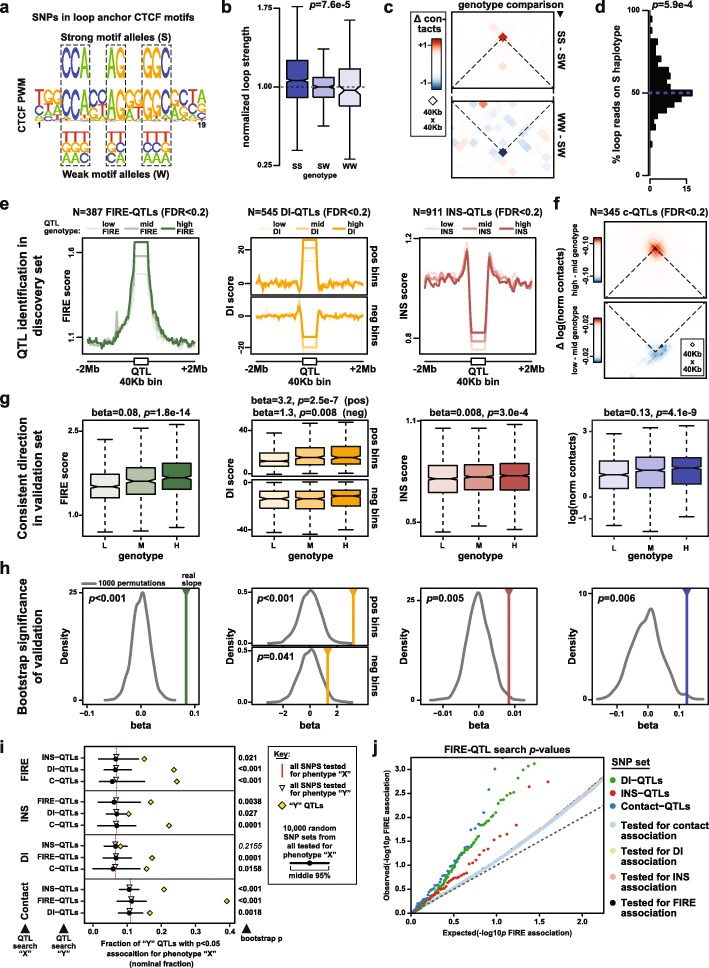
A genetic contribution to variations in 3D chromatin conformation. **a** A CTCF Position Weight Matrix (PWM) is shown (Jaspar MA0139.1). Eight positions boxed by dashed lines have probability > 0.75 for a single base. We refer to SNPs at these positions as “motif disrupting SNPs.” Alleles matching the consensus base in the motif are labeled “strong motif alleles (S),” and alleles matching any other base are labeled “weak motif alleles (W).” **b** Boxplot shows the distribution of interaction frequencies at loops with exactly one anchor containing a CTCF motif disrupting SNP (*N* = 138), separated according to genotype. For each SNP, loop strengths are normalized to the mean value of the heterozygous genotype (WS). There is significant linear relationship between normalized loop strength and genotype by linear regression (*p =* 7.6e-5). **c** Aggregate contact map shows the average difference in interaction frequency per loop between SS and SW genotypes (top; *N* = 117 SNPs), and between SW and WW genotypes (bottom; *N* = 31 SNPs). The cross point of dotted lines indicates the 40-Kb bin containing the loop being evaluated. **d** Histogram shows the allelic imbalance in reads connecting loop anchors on the S vs W haplotypes in WS heterozygotes (*N* = 135 loops). The mean percentage of reads on the S haplotypes is significantly larger than 0.5 (*p =* 5.9e−4 by one-sided *t* test). **e** Line plots show signal of FIRE-QTL, INS-QTL, and DI-QTL by QTL genotype using 11 independent YRI individuals. Each plot shows the indicated phenotype as lines with light color, medium color, and dark color representing average signal across LCLs with the low signal genotype, medium signal genotype, and high signal genotype, respectively. For DI-QTLs, we split all 40-Kb QTL bins into two groups, based on the presence of either upstream DI bias (upper panel) or downstream DI bias (bottom panel). **f** For C-QTLs, an aggregate contact plot analogous to panel c is used to show the average difference in BNBC corrected interaction frequency (“Δ log(norm contacts)”) between the high and medium contact genotypes (top; N = 138 interactions), and between the genotypes medium and low genotypes (bottom; *N* = 94 interactions). The cross point of dotted lines indicates the 40-Kb test bin in question. **g** Boxplots show signal by QTL genotype using additional 6 individuals as a validation set. In each boxplot, three boxes with light shade, medium shade, and dark shade represent the average signal in the 40-Kb QTL bin from individuals with the low signal genotype, medium signal genotype, and high signal genotype, respectively. **h** Results of permutation test to evaluate the statistical significance of results in **g**. The solid vertical lines show the linear regression slope values obtained from the validation set (*N* = 6 individuals). The gray curves show the distributions of slope values obtained from 1000 random permutations. Corresponding bootstrap *p* values indicated in the upper left corner of each subpanel. **i** Line plot shows the fraction of “Y” QTL SNPs with nominal significance (*p <* 0.5) when tested for association with phenotype “X” (“nominal fraction”). Yellow diamonds show nominal fractions for phenotype “Y” QTLs. Red dashed lines show nominal fractions for all SNPs tested for association with phenotype “X,” and open triangles show nominal fractions for all SNPs tested for association with phenotype “Y.” Black circles and lines indicate the median and middle 95% range of 10,000 permutations in which SNPs were selected at random from the phenotype “Y” test SNPs. The number to the right of each line indicates the bootstrap *p* value (fraction of permutations with a nominal fraction higher than observed for “Y” QTLs). **j** QQ plot shows FIRE-QTL search results, including all SNPs tested for FIRE association (black points, *N* = 128,137), and several subsets of FIRE-QTL test SNPs as follows: SNPs also tested for DI association (light green, *N* = 46,784), SNPs also tested for INS association (light red; *N* = 6238), SNPs also tested for contact frequency association (light blue; *N* = 69,847), DI-QTLs (dark green, *N* = 152), INS-QTLs (dark red, *N* = 60), C-QTLs (dark blue, *N* = 53)

To examine genetic influence on 3D chromatin conformation, we first considered genetic variants overlapping CTCF motifs at chromatin loop anchors [14], because disruption of these CTCF motifs by genome engineering has been shown to alter chromatin looping [23]. We focused on SNPs at key positions in anchor CTCF motifs (“motifs disrupting SNPs,” defined as SNPs in positions in the CTCF Position Weight Matrix (PWM) where a single base has a probability of > 0.75, Fig. 4a). We observed a significant linear relationship between SNP genotype and the strength of corresponding loops (*p =* 7.6e−5 by linear regression; Fig. 4b, c). We also examined whether individuals heterozygous for anchor disrupting SNPs showed allelic imbalance in loop strength. To facilitate this analysis, we used the HaploSeq [43] method to generate chromosome-span haplotype blocks for each LCL (Additional file 6: Table S7). Although few Hi-C read pairs overlap a SNP allowing haplotype assignment (mean 7.89% of usable reads per LCL), we did observe that the haplotype bearing the stronger motif allele tends to show more reads connecting the corresponding loop anchors (*p =* 5.9e−4 by one-sided *t* test of mean > 0.5; Fig. 4d). Our observation that CTCF motif SNPs can modulate 3D chromatin conformation is consistent with similar findings reported from ChIA-PET data [53] and a recent report of haplotype-associated chromatin loops published while this manuscript was in preparation [27].

Motivated by these preliminary observations of genetic effects on 3D chromatin conformation, we next searched directly for QTLs associated with Hi-C-derived features of 3D chromatin conformation. Power calculations indicated that, despite limited sample size, we were moderately powered to find QTLs with strong effect sizes using a linear mixed effects model (LMM) approach that incorporates Hi-C replicates for each LCL (Additional file 7: Table S8). We conducted a targeted search for QTLs associated with variation in FIRE, DI, INS, and contact frequency. We did not include PC1 in the QTL search because we reasoned that individual genetic variants would be more likely to have detectable effects on local chromatin conformation rather than large-scale features like compartmentalization. For this same reason, we used modified versions of DI scores and INS calculated with a window size of 200 Kb upstream and downstream of the target bin, rather than the standard 2-Mb window size for DI [16] or 480-Kb window size for INS [8]. We also limited our QTL searches to the 11 unrelated YRI individuals in our study (referred to below as the “discovery set”) to mitigate potential confounding effects of differences between populations.

For each 3D genome phenotype under study, we identified a list of testable bins that showed appreciable levels of signal in at least one individual in our discovery set (see the “Identification of QTLs” section for full description of test bin and SNP selection). We also identified a unified set of test SNPs with at most one tag SNP among those in perfect LD in each 40-Kb bin. For each testable bin, we measured the association of the given 3D chromatin phenotype with all test SNPs in that bin. In cases where multiple SNPs in the same bin were associated with the phenotype, we selected only the most significantly associated SNP per bin for our final QTL list. Ultimately, at FDR < 0.2, we identified 387 FIRE-QTLs (i.e., testable bins in which FIRE score is associated with at least one SNPs in that bin; comprising 6.6% of tested bins), 545 DI-QTLs (4.2% of tested bins), and 911 INS-QTLs (12.0% of tested bins) (Fig. 4e, Additional file 1: Figure S9a, Additional file 8: Table S9). For analysis of DI-QTLs, we separated the testable bins into those with upstream bias and those with downstream bias (see the “FIRE, DI, and INS QTL searches” section), because we observed a “Simpson’s paradox”, which occurs when a trend present in different groups of data disappears when all data is combined (Additional file 1: Figure S9b).

We also searched for QTLs associated directly with interaction frequency in individual contact matrix cells using an LMM approach like that described above for FIRE, DI, and INS. The large number of cells in a Hi-C contact matrix, together with limited sample size, made a true genome-wide QTL search impractical. Thus, we limited our QTL search for contact matrix QTLs (“C-QTLs”) to matrix cells that showed significant biological variability in our samples, as described above. We tested for association in our discovery set between the BNBC-normalized interaction frequency in these variable matrix cells and the genotype of test SNPs in either of the two anchor bins. We selected at most one QTL SNP per matrix cell, using association *p* value to prioritize, finally yielding 345 C-QTL SNPs associated with 463 matrix cells at an IHW-FDR threshold of 0.2 (Fig. 4f, Additional file 1: Figure S9d, Additional file 9: Table S9).

To evaluate the reproducibility of each of these QTLs sets (FIRE-QTLs, DI-QTLs, INS-QTLs, and C-QTLs), we examined Hi-C data from six individuals who were not included in our discovery set (we refer to these six individuals as our “validation set”; Additional file 2: Table S2). These individuals come from four different populations (CEU, PUR, CHS, YRI) and include a child of two individuals in the discovery set (YRI-13/NA19240 is a child of YRI-11/NA19238 and YRI-12/NA19239). In each case, we found a significant linear relationship in the validation set between QTL genotype and the corresponding 3D chromatin phenotype (*p =* 1.8e−14 for FIRE-QTLs, *p =* 2.5e−7 for DI-QTLs at positive DI bins, *p =* 0.008 for DI-QTLs at negative DI bins, *p =* 3e−4 for INS-QTLs, *p =* 4.1e−9 for C-QTLs; Fig. 4g). To provide an additional and more stringent estimate of the significance of these observations, we performed permutations by randomly selecting sets of test SNPs and measuring the linear relationship between genotype and phenotype in the validation set. In all cases, the observed relationship was also significant by this more conservative bootstrap approach (*p <* 0.001 for FIRE-QTLs, *p <* 0.001 for DI-QTLs at positive DI bins, *p =* 0.041 for DI-QTLs at negative DI bins, *p =* 0.005 for INS-QTLs, *p =* 0.006 for C-QTLs; Fig. 4h). We also found that the linear relationship between genotype and phenotype at these QTLs is generally consistent with Hi-C data analyzed at a variety of resolutions (i.e., bin sizes), and when the data is normalized with Knight-Ruiz (KR) matrix balancing [14] instead of the HiCNorm method we used for the rest of our analysis (Additional file 1: Figure S9c).

There is little direct overlap between our different QTL sets (Additional file 1: Figure S9e), likely due to limited power and different testable bins for each metric. However, we observed genotype-dependent INS at FIRE-QTLs and C-QTLs and genotype-dependent FIRE score at INS-QTLs and DI-QTLs (Additional file 1: Figure S10a), suggesting that overlapping signal between different types of 3D chromatin QTLs might exist below the level of genome-wide significance. To more rigorously assess overlapping signal between our QTL sets, we examined shared association below the threshold of multiple test correction, inspired by similar approaches reported elsewhere [54]. Our underlying assumption in this analysis is that genetic association studies of two different phenotypes “*X*” and “*Y*” with overlapping (or partially overlapping) genetic architecture may have few direct QTL overlaps due to limited power or different study designs, but the shared signal should become apparent when results below the level of genome-wide significance are considered. We thus calculated the fraction of QTLs for a given phenotype *X* that exceed a nominal level of significance (*p* < 0.05) when tested for association with a different phenotype *Y*. We refer to this value as the “nominal fraction” below and in Fig. 4i. To assign a level of statistical significance to these nominal fractions, we approximated the null distribution by calculating nominal fractions for 10,000 sets of SNPs selected randomly from among all test SNPs. In almost all pairwise comparisons between 3D chromatin QTL types examined here, we found that the observed nominal fractions are significantly higher than fractions that would be expected in the absence of shared genetic architecture (Fig. 4i, j).

### Contribution of 3D chromatin QTLs to molecular phenotypes and disease risk

**Fig. 5:**
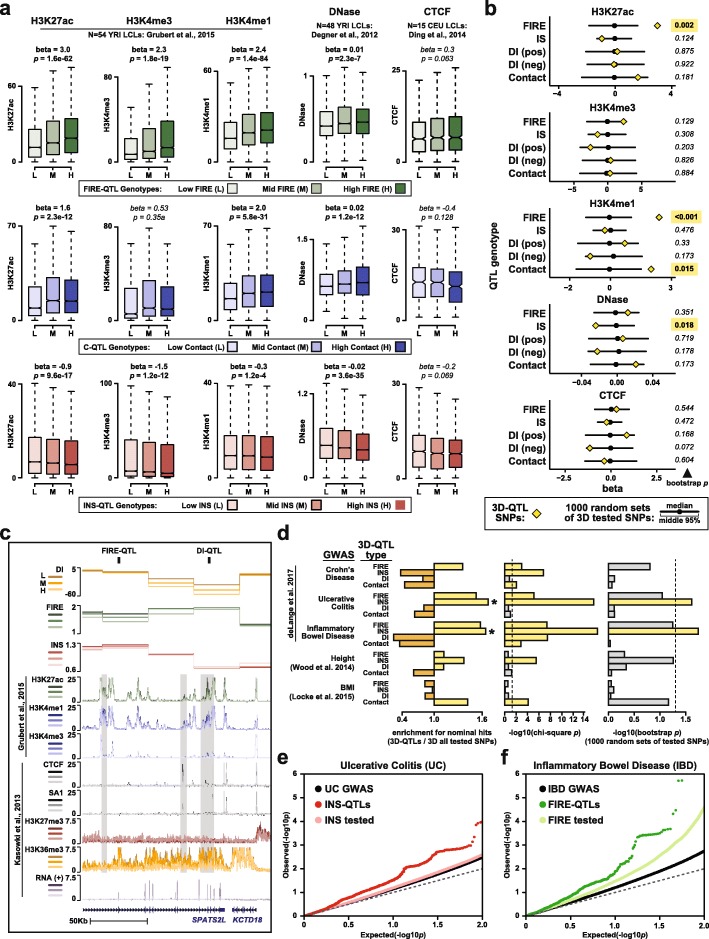
Contribution of 3D chromatin QTLs to other molecular and organismal phenotypes. **a** Boxplots show signal for epigenetic phenotypes separated by genotype at FIRE-QTLs (top row), C-QTLs (middle row), and INS-QTLs (bottom row). Epigenetic signals averaged across all peaks in 40-Kb bin. Linear regression *p* and beta values shown above each plot. *p* value < 0.05 in bold, others in italics. **b** Line plots shows beta values of linear relationships between QTL genotypes indicated to the left and epigenetic phenotype indicated above each subpanel. Yellow diamonds show beta values for the true QTLs sets as shown in (a) or Additional file 1: Figure S10b. Black circles and lines indicate the median and middle 95% range, respectively, of 1000 permutations in which SNPs were selected from those tested in the QTL search indicated to the left of each line. The number to the right of each line indicates the bootstrap *p* value (fraction of permutations with abs(beta) higher than observed for the true QTL set). Yellow boxes highlight values < 0.05. **c** Genome browser view (chr2:201,222,342-201,386,844; hg19) showing examples of a DI-QTL (chr2:201333312) and FIRE-QTL (chr2:201254049). All signals plotted as a function of DI-QTL genotype (L = Low DI, M = medium DI, H=High DI). Gray boxes highlight regions where epigenetic signals stratify by DI-QTL genotype. **d** Left subpanel shows the enrichment values for 3D QTL SNPs with nominal significance in the indicated GWAS study calculated as follows: (fraction of indicated 3D QTL SNPs with nominal significance in the indicated GWAS)/(fraction of 3D *test* SNPs with nominal significance in the indicated GWAS). Asterisks mark values with *p <* 0.05 by chi-square test (middle panel), and permutation test (right panel). Right panel shows the proportion of 1000 random SNP subsets (selected from the tested SNPs) with enrichment values higher than the indicated true QTL set. Dotted lines mark *p <* 0.05. **e** QQ plot shows the results of UC GWAS with all tested SNPs shows as black points, and two subsets as follows: SNPs also tested in our INS-QTL search (light red), and SNP called as INS-QTLs or in perfect LD with INS-QTLs in the same 40-Kb bin (dark red). **f** QQ plot shows the results of IBD GWAS with all tested SNPs shows as black points, and two subsets as follows: SNPs also tested in our FIRE-QTL search (light green), and SNP called as FIRE-QTLs or in perfect LD with FIRE-QTLs in the same 40-Kb bin (dark green)

Given the correlation observed between 3D chromatin variation and epigenome variation, we next investigated whether 3D chromatin QTLs could modulate both the epigenome and 3D genome. Here, we used published ChIP-seq data for histone modifications (H3K4me1, H3K4me3, H3K27ac) in a large set of 65 YRI LCLs [39], DNase-seq data from 59 YRI LCLs [38], and CTCF ChIP-seq data from 15 CEU LCLs [55]. In many cases, we found a significant linear relationship between 3D chromatin QTL genotypes and these different epigenetic phenotypes, even when only considering individuals in these datasets were not included in our QTL discovery or validation sets (Fig. 5a, Additional file 1: Figure S10b; 54/65 individual for histone modification ChIP-seq, 48/59 for DNase-seq, and all 15/15 for CTCF ChIP-seq). For example, at FIRE-QTLs, the high-FIRE allele is also associated with higher levels of active histone modifications and chromatin accessibility (Fig. 5a). We note that although these associations are all significant by linear regression, only H3K27ac and H3K4me1 passed more conservative permutation testing in which the null distribution is approximated by selecting random SNPs from the full set of tested SNPs (Fig. 5b). At INS-QTLs, the slope of these genotype-phenotype relationships is inverted such that higher levels of histone modifications and chromatin accessibility are associated with the low INS score allele (i.e., more contact insulation), although only the association with chromatin accessibility is significant by both linear regression and permutation test (*p =* 3.6e−35 by linear regression, bootstrap *p =* 0.018; Additional file 1: Figure S10b,d). The genotype-phenotype relationships observed at DI-QTLs are not as clear as for other metrics (Fig. 5b, Additional file 1: Figure S10a), but this may be expected because increased histone modifications or chromatin accessibility can influence DI in either direction, potentially confounding this type of aggregate analysis. Anecdotally, we did observe examples of individual DI-QTLs where genotype appears to correlate with epigenomic phenotype (Fig. 5c). At C-QTLs, the high-contact alleles show higher levels of the enhancer-associated mark H3K4me1 in the two anchor bins that connect the corresponding matrix cell. Moreover, the fraction of C-QTLs with *p <* 0.05 association with H3K4me1 in a published set of H3K4me1-QTLs is significantly higher than expected in the absence of shared genetic association (*p =* 6.9e−6 by chi-square test, bootstrap *p =* 0.028; Additional file 1: Figure S10c, d).

Finally, we sought to examine whether 3D chromatin QTLs might contribute risk for complex diseases. There are 44 direct overlaps between our 3D chromatin QTLs (or SNPs in perfect LD in the same 40-Kb bin) and NHGRI-EBI GWAS catalog [56] (Additional file 9: Table S10). However, the significance of these direct overlaps is hard to assess given the differences between the populations and study designs. Thus, we examined overlaps below the level of genome-wide significance by looking at “nominal fractions” (described above) to assess the shared signal between association studies. We compiled full summary statistics for large GWAS (> 50,000 individuals) of immune-relevant phenotypes including Crohn’s disease (CD), ulcerative colitis (UC), and inflammatory bowel disease (IBD) [57], as well as studies of the non-immune phenotypes height [58] and body mass index (BMI) [59]. We observed striking enrichments for INS-QTLs among variants with nominal associations for UC and IBD risk (1.67- and 1.65-fold, respectively), and these enrichments are significant by both chi-square and permutation tests (INS-QTL with UC chi-square *p =* 2.5e−16 and bootstrap *p =* 0.024; INS-QTL with IBD chi-square *p =* 5.5e−17 and bootstrap *p =* 0.018; Fig. 5d, e). We also note a trend in which FIRE-QTLs show nominal association with UC and IBD (1.36- and 1.58-fold enrichment, respectively), although these observations fall just below the threshold of significance by the more stringent permutation test (FIRE-QTL with UC chi-square *p =* 7.6e−6 and bootstrap *p =* 0.090; FIRE-QTL with IBD chi-square *p =* 4.2e−8 and bootstrap *p =* 0.056; Fig. 5d, f).

## Discussion

**Fig. 6:**
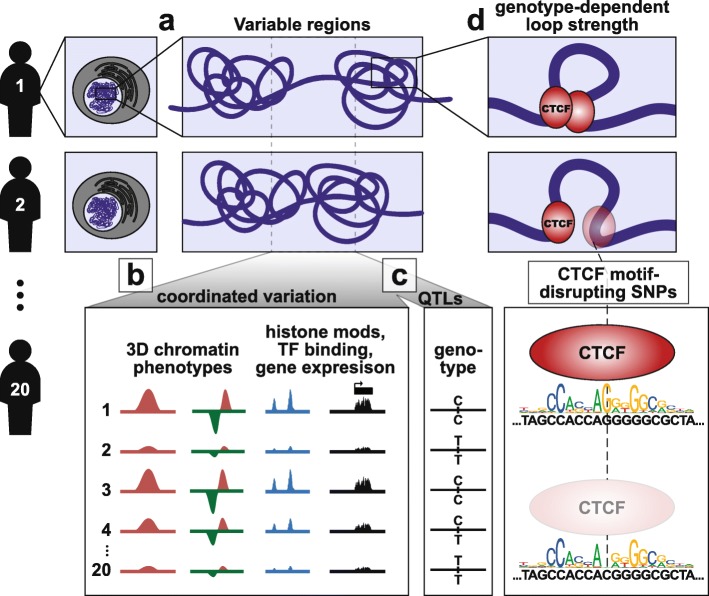
Summary of findings related to 3D chromatin variability and genetic influence. **a** There are thousands of regions that vary between individuals in one or more features of 3D chromatin conformation. **b** These regions tend to vary across individuals in multiple 3D chromatin features as well as in histone modifications, TF binding, and gene expression. **c** We identify hundreds of QTLs associated with 3D chromatin variation at a subset of these variable regions. **d** SNPs that disrupt CTCF binding motifs modulate chromatin loop strength

Our results provide the first systematic characterization of chromatin conformation variation across unrelated individuals at the population level (Fig. 6). The most important finding of our study is that genetic variation influences multiple features of 3D chromatin conformation, and does so to an extent that is detectable even with limited sample size and Hi-C resolution. To the best of our knowledge, this represents the first report of QTLs directly associated with 3D chromatin conformation. However, there are limitations to our QTL search that are important to note here. First, the small sample size means that our power to detect QTLs is limited, and thus, our QTL sets should not be considered comprehensive, even within the targeted regions over which the QTL searches were performed. Second, in order to identify QTL sets that could be analyzed in aggregate, we tolerated elevated type I error by using an FDR threshold of 0.2 (as done previously for molecular QTL studies with limited power [40]). While the QTL sets reported here likely contain false positives, the abundance of true positives is suggested by aggregate analyses showing that the genotype-phenotype relationships are reproduced in an independent set of six individuals, and consistent genotype-phenotype relationships at 3D QTLs are apparent in orthogonal epigenomic datasets generated independently by other labs on material isolated independently, and from different LCLs/individuals.

Another key finding of our study is that regions which vary in 3D chromatin conformation between individuals also tend to vary in measures of transcriptional and regulatory activity. This supports the existence of shared mechanisms that underlie variation in 3D chromatin conformation, transcription, and epigenomic properties. Our data does not resolve whether variation in higher-order chromatin conformation leads to variation in gene expression and histone modification state, or vice versa. However, we suspect that no single mechanism or causal hierarchy applies to all regions of the genome with variation in one or more of these molecular phenotypes. Our QTL results suggest that, in at least some cases, genetic variation is likely the underlying mechanism that leads to the observed multi-omic variation. This raises the question of whether 3D chromatin QTLs are fundamentally the same as QTLs previously described for other molecular phenotypes (e.g., eQTLs, dsQTLs, histoneQTLs; collectively referred to below as “molQTLs”) or, rather, represent a separate set of QTLs not detectable with other methods. This question is difficult to answer in the present study for two main reasons: (1) Our power is limited, and thus, we cannot say with confidence that a given SNP is *not* a 3D chromatin QTL. Many molQTL studies also have limited power and are thus also prone to high type II error (i.e., false negatives). (2) Our QTL searches, like most molQTL studies, are not truly genome-wide because testable regions and testable SNPs are pre-selected to limit the search space. These selection criteria can differ widely across studies, making direct QTL-to-QTL comparisons challenging. The observation of genotype-dependent epigenetic signal at 3D chromatin QTLs suggests that at least some 3D chromatin QTLs could also be detected as other types of molQTLs if those studies had enough statistical power. However, the limited overlap between 3D chromatin QTLs and published molQTLs (even when considering SNPs with only a nominal level of significance) suggests that the QTLs with largest effects on 3D chromatin conformation are not necessarily the same as those with large effects on other molecular phenotypes, and vice versa. Therefore, it is likely that QTL studies directed toward different types of molecular phenotypes (including 3D chromatin features) are likely to be complimentary rather than redundant.

We expect that future studies with higher resolution Hi-C data and larger sample size will be important to identify functional variants modulating 3D chromatin conformation, and to further dissect the mechanistic relationships between genetics, 3D chromatin conformation, and other molecular phenotypes. We anticipate that these studies will continue to reveal cases in which perturbation of 3D chromatin conformation is a molecular mechanism through which disease-associated genetic variants confer disease risk. The present study provides initial discoveries of genetic influence on 3D chromatin conformation and an analytical framework that we believe will facilitate future efforts to unravel the molecular basis of genetic disease risk.

## Methods

### Hi-C data generation

Hi-C was performed as previously described [13]. We note that all Hi-C experiments were performed using a “dilution” HindIII protocol, rather than the newer “in situ” version of the protocol, for consistency because data generation began before the invention of in situ Hi-C. In addition, the resolution of 40 Kb used here for most analysis was determined primarily by sequencing depth rather than choice of a restriction enzyme. Thus, even if a 4-cutter like MboI had been used, the prohibitive cost of sequencing would have prevented us taking advantage of the additional possible resolution.

### Hi-C data processing

#### Alignment with WASP

Read ends were aligned to the hg19 reference genome using BWA-MEM [60] v0.7.8 as single-end reads with the following parameters: -L 13,13. We used the WASP pipeline [40, 45] to control for potential allelic mapping biases, with some modifications to account for unique aspects of Hi-C data: BWA-MEM can produce split alignments where different parts of a read are aligned to different parts of the genome. This is critical for Hi-C data, because a read can span a Hi-C ligation junction between two interacting fragments. In the case of a split alignment, BWA-MEM will mark the higher-scoring alignment as the primary alignment. For Hi-C data, this is not ideal—we want the five-prime-most alignment (before the ligation junction) to be the primary alignment. To account for this, we further processed the alignments from BWA-MEM to select the five-prime-most alignment in cases where one read was split. Reads without an alignment to the five-prime end of the read were filtered out, as were alignments with low mapping quality (< 10). The WASP pipeline was then used to generate alternative reads by flipping the allele in reads overlapping SNPs, and these reads were then realigned using the same pipeline. As input to WASP, we included all SNPs and indels present in the PUR individuals in our set (HG00731, 732, 733), all CHS individuals in 1000 genomes (we included all CHS to account for the fact that no 1000 genomes genotype calls were available for HG00514), all YRI individuals in 1000 genomes (we included all YRI individuals to account for the fact that no 1000 genomes genotype calls were available for GM19193), and the H1 cell line [21] (to facilitate uniform processing and comparisons between LCLs and H1-derived datasets). After alignment of the alternative reads, alignment locations of the original reads and alternative reads were compared (using WASP), and only the original reads for which all alternative reads aligned at the same location with same CIGAR string were kept. Reads overlapping indels were removed. Reads were then re-paired, and only pairs in which both reads survived this filtering were kept. PCR duplicates were removed using Picard tool (http://broadinstitute.github.io/picard/) with default parameters. To ensure that our adapted WASP pipeline removed allelic mapping biases effectively, we simulated all possible 100-bp single-end reads spanning SNPs in our LCLs for chromosome 22 and aligned them back to the genome using our adapted WASP pipeline. We found that no SNPs depart from 50/50 mapping ratio between reference and alternative allele in these simulations.

We also took steps to remove any potential artifacts due to HindIII polymorphisms. Hi-C data was obtained by cutting the genome with HindIII, so we reasoned that SNPs or indels that disrupt existing HindIII sites or create novel HindIII sites could lead to differential cutting of two alleles and thus the appearance of differential contact frequency. To mitigate these potential artifacts, we identified all HindIII sites that would be disrupted or created by genetic variants present in our samples, and removed all reads within 1 Kb of these polymorphisms in all individuals.

#### Contact matrix calculations

Matrices were generated and normalized as previously described [21, 61]. Briefly, intra-chromosomal read pairs were divided into 40-Kb bin pairs based on five-prime positions. The number of read pairs connecting each pair of 40-Kb bins was tallied to produce contact matrices for each chromosome. Raw counts in the contact matrices were then normalized using HiCNorm [61] to correct for known sources of bias in Hi-C contact matrices (GC content, mappability, fragment length) [62]. Bins that are unmappable (effective fragment length, GC content, or mappability is 0) were assigned NA values. These normalized matrices were further quantile normalized across samples to account for differing read depths and mitigate potential batch effects. One such quantile normalized matrix was generated for each chromosome in each replicate, as well as in each sample (replicates pooled together). We eliminated chromosomes X and Y from all downstream analyses due to the gender differences between our samples. For KR norm matrices used in Additional file 1: Figure S9, we used the Juicer pipeline to generate matrices and performed normalization using Juicebox tools [63].

#### PC1 score

PC1 scores were computed using methods defined previously [13]. Briefly, quantile normalized matrices for each chromosome were transformed to observed/expected (O/E) matrices by dividing each entry in the matrix by the expected contact frequency between regions in that matrix at a given genomic distance. For a given matrix, the expected contact frequencies were computed by averaging contact frequencies at the same distance in that each matrix. The O/E matrices were further transformed to Pearson correlation matrices by the “cor” function in R and eigen vectors (principal components) were computed using the “cov” function in R. Generally, the first eigenvector (“PC1”) reflects A/B compartmentalization. However, for some chromosomes, we have seen that the second or third eigenvector sometimes reflects compartmentalization, while the first eigenvector reflects other features like the two chromosome arms. To identify such cases, we examined the first three eigenvectors for each chromosome in each replicate by calculating their correlation with the gene density, because the true compartmentalization pattern is correlated with gene density, while other properties like chromosome arms are not. We required that PC1 show the highest correlation with gene density among the first three eigenvectors in every replicate. If this was not the case for a given chromosome, we eliminated that chromosome from all downstream PC1 analyses in all individuals. Six chromosomes were eliminated in this way: chr1, chr9, chr14, chr19, chr21, and chr22. For the chromosomes that passed this filter, the sign of the first eigenvector, which is arbitrary, was adjusted such that positive PC1 values correspond to compartment A (higher gene density). We note that gene density was not used in the actual PCA calculations, but was only used to orient the otherwise arbitrary direction of PC1, and to systematically eliminate problematic chromosomes where we could not be sure that the first eigenvector captures compartmentalization as opposed to other chromosome features.

#### Directionality index

Directionality index was computed as previously described [16]. Briefly, upstream and downstream contacts within 2-Mb window for each 40-Kb bin were counted, and chi-square statistics were calculated under equal assumption. The sign of the chi-square statistics was adjusted such that positive values represent upstream bias. For some bins, there are more than five NA bins within 2-Mb window, and DI for those bins are not calculated. As noted in the main text, we made a slight variation of these DI scores for the QTL searches in which DI was recalculated using a window size of 200 Kb to capture more local features.

#### Insulation score

Insulation scores were computed as previously described [7] with some adjustments. Briefly, contacts linking upstream and downstream 400-Kb windows for each 40-Kb bin were calculated in the O/E matrices instead of raw matrices. We further divided the contact frequency by the average of upstream and downstream 400-Kb windows, to account for differences in contact density across the chromosome. The insulation scores were then ranged from 0 to 1, representing absolute insulation and no insulation respectively. Insulation scores for bins in which more than 50% of matrix cells in the 400-Kb window have NA values were not computed. For the QTL search, we re-calculated insulation scores using 200-Kb window.

#### TADs calling

TADs were called using the same approach as described previously [16]. DI values for each 40-Kb bins were used to build a Hidden Markov Model and predict the probability upstream bias, no bias, and downstream bias. Regions switching from upstream bias to downstream bias were called as boundaries.

#### FIRE

We first calculated FIRE score for each of 20 individuals, as described in our previous study [19]. Specifically, we mapped the raw reads to the reference genome hg19 as described above. Next, we removed all intra-chromosomal reads within 15 Kb and created 40-Kb raw Hi-C contact matrix for each individual for each autosome. For each 40-Kb bin, we calculated the total number of intra-chromosomal reads in the distance range of 15–200 Kb. We then filtered bins as follows, starting from 72,036 autosomal 40-Kb bins: First, we removed 40-Kb bins with zero effective fragment size, zero GC content, or zero mappability score [61]. Next, we filtered out 40-Kb bins within 200 Kb of the bins removed in the previous step. We further filtered out 40-Kb bins overlapping with the chr6 MHC region (chr6:28,477,797-33,448,354; hg19), which has extremely high SNP density that can make it difficult to correct for allelic mapping artifacts. This left 64,222 40-Kb bins for downstream analysis. Next, we applied HiCNormCis [19] to remove systematic biases from local genomic features, including effective fragment size, GC content, and mappability. The normalized total number of cis intra-chromosomal reads is defined as FIRE score. We further performed quantile normalization across multiple individuals using R package “preprocessCore.” The final FIRE score is log transformation log2(FIRE score + 1) and converted into a *Z*-score to create a mean of 0 and standard deviation of one. To identify significant FIRE bins in each individual, we used one-sided *p* value < 0.05. Ultimately, merging across all individuals, we identified 6980 40-Kb bins which are FIRE bin in at least one of 12 YRI individuals. Consistent with our previous findings [19], we observed significant enrichment of GM12878 typical enhancers and super enhancers among these 6980 40-Kb FIRE bins (Additional file 1: Figure S1d). GREAT analysis [64] further showed immune-related biological pathways and disease ontologies are enriched in these 6980 40-Kb FIRE bins (Additional file 1: Figure S1e).

### Comparison of intra-individual vs inter-individual variation

To estimate variability between replicates, we computed the Pearson correlation coefficient for all pairs of replicates for each score (DI, INS, FIRE, and PC1). Replicate pairs then can be divided into two groups based on whether they are from the same individuals or different individuals, as illustrated in Additional file 1: Figure S4c. We then tested if the distribution of Pearson correlation coefficients were different comparing two groups. Similar analysis was performed for contact matrices. For contact matrices, we calculated the Pearson correlation coefficient for each distance and each chromosome separately as shown in Fig. 1c.

### Variable regions

#### *F* test for variable bins

To identify regions that are variable across genomes, we used a linear normal model with an empirical Bayes variance estimator using the “eBayes” in limma [48] function with default parameters. First, values for each 40-Kb bin in hg19 reference genome were calculated for each metric tested (DI, FIRE, INS, PC1) as described above. DI, PC1, and INS scores were calculated based on contact matrices quantile normalized across 40 replicates. FIRE scores were calculated based on raw counts using HiCNormCis [19] and then quantile normalized across 40 replicates. Second, we filtered out bins that are not testable. Specifically, FIRE scores were only tested for bins that are FIRE regions (*p* < 0.05) in any of 40 replicates. DI scores were only tested for bins where strong biases are observed (abs(DI) > 10.82757, which correspond to chi-squared test *p* value 0.001) in any of 40 replicates. INS were only tested for bins where strong insulation is observed (*z*-score transformed INS score < − 1) in any of 40 replicates. No similar filterers were performed for PC1 scores. Third, we filtered out any bins that overlapping large SVs (> 10,000 bp) to avoid effect caused by SVs. Specifically, for FIRE, INS, and DI scores, bins that are within 200 Kb, 400 Kb, and 2 Mb respectively upstream or downstream of large SVs were removed. For PC1 scores, bins directly overlapping large SVs were removed. Lastly, we applied limma standard model with individual as a fixed factor and eBayes correction. To estimate empirical false-positive rate (FDR), we bootstrapped replicates to calculate the number of false positives in random background. Briefly, we randomly selected 40 or 22 replicates with replacement for LCL20 and YRI11 respectively and identified variable regions as mentioned above. We performed 1000 permutations and calculated empirical FDR as the average positive hits in 1000 permutations divided by number of hits in real data.

#### Normalizing Hi-C contact matrices using BNBC normalization

To directly compare individual Hi-C contact matrix cells across samples, we sought to remove unwanted per-matrix-cell variation owing to date of processing or other unknown “batch” effects. To this end, we developed Bandwise Normalization and Batch effect Correction (BNBC), described and evaluated in a separate manuscript (preprint on bioRxiv https://www.biorxiv.org/content/10.1101/214361v1). A brief description follows. For each chromosome and for each strata of distance between loci (a matrix “band,” hence the term “bandwise”), we correct for unwanted variation by taking the log counts-per-million-transformed values of all samples and generating a matrix whose entries are the observations for that chromosome’s matrix band across all samples (columns indexes samples and rows indexes contact matrix cells with anchor bins separated by a fixed distance). We then quantile normalize this matrix and regress out the impact of known batches (here, date of processing) using ComBat [65] (specifically we correct both mean and variance). This procedure essentially conditions on genomic distance. We correct the majority of each contact matrix for each chromosome for each sample: we correct all but the 8 most distal matrix bands, for which we set all values to 0. The choice of the last 8 bands is empirical and reflects the small number of observations in each band matrix. The procedure is implemented in the bnbc package available through Bioconductor (http://www.bioconductor.org/packages/bnbc). Correction of contact matrices was performed on replicate-level data using the following LCLs: GM18486 (YRI-1), GM18505 (YRI-2), GM18507 (YRI-3), GM18508 (YRI-4), GM18516 (YRI-5), GM18522 (YRI-6), GM19099 (YRI-7), GM19141 (YRI-8), GM19204 (YRI-10), GM19238 (YRI-11), GM19239 (YRI-12), GM19240 (YRI-13), HG00731 (PUR-1), HG00732 (PUR-2), HG00512 (CHS-1), HG00513 (CHS-2). We note that NA19239 (YRI-12) replicate 1 and NA19240 (YRI-13) replicate 2 were excluded because the BNBC algorithm requires multiple samples from a given experimental batch to estimate batch effect parameters.

#### Identifying biological variability in Hi-C contact matrices

To identify contacts with significant levels of between-individual variability, we employed the following procedure, which mimics the analysis for INS, DI, FIRE, and PC1, on contact matrices normalized by BNBC (see the “Normalizing Hi-C contact matrices using BNBC normalization” section). For each contact matrix cell (representing loci separated by less than 28 Mb, this is a subset of the matrix cells normalized by BNBC), we used a linear model with individual modeled as a fixed factor, note we have 2 growth replicates for almost every individual. We used a parametric likelihood ratio test (equivalent to an *F* test) to test whether there was significant between-individual variation. We used the IHW framework [50] with the distance between anchor bins as informative covariate, to increase power and estimate false discovery rate. We used a FDR of 10% as significance threshold, resulting in 115,817 contact matrix cells with significant biological variability across the autosomes. To estimate effect size (depicted in Figs. 2a and 3a and Additional file 1: Figure S5), we used a linear mixed effects model with individual as random effect, to decompose the variance into between-individual variability (biological) and within-individual variability (technical). As the measure of biological variability in these figures, we used the estimated biological variance. For this analysis, all 16 samples we normalized using BNBC were used.

#### Correlation with other datasets

To examine correlation between 3D genome organization and other genome features, we re-identified variable regions with the same pipeline mentioned above using only individuals for which data is available for other genome features, and then computed the Spearman correlation coefficient between 3D genome metrics (DI, INS, PC1, and FIRE) and other genome features (RNA-seq, ChIP-seq, and DNase-seq) for each variabel 40-Kb bin. Signals for each 40-Kb bins were calculated by averaging signals for the bin. Specifically, signals for ChIP-seq were the average signal of all peaks within the bin, signals for RNA-seq were the average FPKM of all genes in the bin, and DNase signals were average signal for each base pair in the bin. In some cases, serval consecutive bins were identified as variable. In these cases, we only kept the bin with strongest signal for other genome features among consecutive bins. To generate random backgrounds, we permutated individual labels for the same set of bins and recomputed Spearman correlation coefficient. Ten thousand such permutations were used to calculate the statistical significance of departure from the null hypothesis in which the median value of true correlation values and permutated correlation values are equal. Similar analysis was performed for variable matrix cells with the following modifications. First, we used the variable matrix cells in the preceding section “Identifying biological variability in Hi-C contact matrices.” Second, to correlate matrix-cell-level contacts with bin-level DNase and ChIP-seq signals, we sued the anchor bins of variable matrix cells. Since each anchor bin may belong to more than one matrix cells, we only used each bin once and selected the matrix cell value with the highest Spearman correlation coefficient. Exactly same approach was performed during permutation to ensure a fair comparison.

### Phasing variants

Phasing of variants was performed based on HaploSeq pipeline [43]. Briefly, (1) variants were filtered to keep only bi-allelic SNPs heterozygous in a given individual; (2) aligned Hi-C bam files were realigned and recalibrated using GATK 3.4.0 [66] based on SNPs in the individual; (3) filtered SNPs and realigned bam files were then used as input to run HAPCUT [67]; (4) results from HAPCUT were further filtered to keep only the largest haplotype block and combined with homozygous alt SNPs as input for imputation using Beagle 4.0 [68] using 1000 Genome Phase 3 data excluding individual to phase as reference panel; (5) results from Beagle were then combined with results of HAPCUT by removing conflicting phased SNPs. For all auto chromosomes except 1 and 9 in 18 out 20 individuals, we were able to obtain a single haplotype block. For chromosome 1 and 9, two arms were phased separately because of large heterochromatin region surrounding centromere. X chromosome was only phased for female individuals. We excluded NA19193 and HG00514 from phasing because of the lack of available genotypes through 1000 genomes at the time of phasing. We evaluated accuracy of phasing in three probands in trios (NA19240, NA12878, HG00733) and found phasing results are of very high accuracy (~ 97.71%). Specifically, we calculated accuracy as percentage of correctly phased variants among total phased variants. Only variants whose transmission from parents can be unambiguously identified were used in calculation of accuracy where at least one parent is homozygous. Detailed statistics for phasing are listed in Additional file 5: Table S6.

### CTCF motif variation and looping strength

GM12878 loops and motif positions were obtained from Rao et al. [14] (GSE63525_GM12878_primary+replicate_HiCCUPS_looplist_with_motifs.txt.gz; *N* = 9448 HiCCUPS loops). We limited our analysis to autosomal *cis* loops in which a CTCF motif in one of the anchor regions overlaps a SNP (*N* = 572). To evaluate the impact of motif disruption, we first identified eight “key” positions in the CTCF PWM (Jaspar MA0139.1) [69] in which a single base has higher than 0.75 probability. We refer to SNPs at these positions in motif occurrences with one allele matching the high-probability base as “motif disrupting SNPs.” We refer to alleles matching the consensus base in the motif as strong motif alleles (S), and alleles matching any other base as weak motif alleles (W). There are *N* = 142 loops with a motif disrupting SNP in a convergently oriented CTCF motif, which we refer to below as testable loops. For each testable loop, we extracted the Hi-C interaction frequency in the loop bin from each LCL and classified as either “WW,” “SW,” or “SS” depending on the individual’s genotype at the corresponding motif disrupting SNP. To enable aggregation of data across different SNPs, we set the mean “SW” interaction frequency for each SNP to 1 and normalized all values for that SNP accordingly. These values are plotted in Fig. 4b. In addition, for each testable loop we extracted a submatrix including the loop bin as well as 15 bins upstream and 15 bins downstream. Submatrices with missing values were discarded. For each SNP, we calculated the mean submatrix for each genotype and then subtracted submatrices to calculate the difference in each matrix cell per “W” allele (i.e., SS-SW and SW-WW). These differences were then averaged across all SNPs and plotted in Fig. 4c. Submatrices with missing values were discarded. For the allelic analysis in S/W heterozygous individuals, we used chromosome-span phasing results (see the “Variable regions” section) to split the Hi-C reads from each chromosome in each LCL into two separate haplotypes. Specifically, we required at least one base pair overlap with phased heterozygous SNPs with high base calling score (> 13) and high mapping quality (> 20). Reads overlapping indels or containing SNPs from both haplotypes were not used. Approximately 7.89% of Hi-C reads covered a heterozygous variant and could thus be assigned to one of the two haplotypes. The accuracy of haplotype assignment was evaluated by fraction of homologous-trans (h-trans) read, which contain SNPs from both haplotypes. On average, ~ 1% reads were h-trans, suggesting high quality of the assignment. For each testable loop, we defined 40-Kb windows around the center of each loop anchor region and calculated the number of reads connecting these two anchor windows (“loop reads”) on each haplotype. For each heterozygous LCL, we then calculated the percentage of loop reads that occur on the haplotype containing the S allele at the motif disrupting SNP anchor. We required that at least 10 total loop reads were present for a given loop in a given heterozygous LCL, leading to a total of 218 data points from 105 different loops for inclusion in Fig. 4d.

### Identification of QTLs

#### Testable bins

To identify testable bins for FIRE-QTL, DI-QTL, INS-QTL, and C-QTL searches, we began with 72,036 autosomal 40-Kb bins based on reference genome hg19. We eliminated “unreliable” bins with effective length, GC content, or mappability equal to zero [62], resulting in 66,597 bins remaining. We further removed any 40-Kb bins within 200 Kb of an unreliable bin, resulting in 64,337 40-Kb bins. We also removed bins covering the chr6 MHC locus (hg19: chr6:28,477,797-33,448,354), which is extremely polymorphic and may lead to complex mapping artifacts that are difficult to correct for. To eliminate false signals in Hi-C data that could arise from large structural variations (SVs), we obtained SVs from the 1000 Genomes consortium [35] (ftp://ftp-trace.ncbi.nih.gov/1000genomes/ftp/phase3/integrated_sv_map/ALL.wgs.integrated_sv_map_v2.20130502.svs.genotypes.vcf.gz) and removed bins which overlap one or more structural variants of any size previously annotated in these individuals (*N* = 123,015 SVs), or within 200 Kb of large structural variations (> 10 Kb, *N* = 1253 SVs). These filtering steps yielded a set of 51,511 testable bins, which represent a common starting point for FIRE-QTL, DI-QTL, INS-QTL, and C-QTL searches as described below.

#### Testable SNPs

We began with a list of 15,765,667 variants among all 20 LCL individuals (Additional file 2: Table S3). We kept 14,177,284 variants among 11 unrelated YRI individuals and removed all indels, HindIII site polymorphisms, multi-allelic SNPs, and SNPs with minor allele frequency (MAF) < 5%. We also required that remaining SNPs were within the 51,511 testable bins described above and that both alleles were present in at least 2 individuals in the discovery set individuals. (*N* = 4,132,791 SNPs remaining). Finally, where multiple SNPs in the same bin were in perfect LD among 11 unrelated YRI individuals, we selected one with the smallest genomic position (to avoid the introduction of a random selection that would not be perfectly reproducible), ultimately yielding 1,304,404 potentially testable SNPs that served as a common input set to all QTL searches.

#### Power calculations

To explore the power of our approach and data, we performed a Monte Carlo-based power calculation. Specifically, we varied four variables: (1) the minor allele frequency of a variant, (2) the effect size of genotype (a fixed effect), (3) the variability between subjects (a random effect), and (4) the variability of the residuals. For contact QTLs, we also varied the mean of the Hi-C contact frequency in question. For analyses reported, we fixed the number of replicates per subject to be 2 (consistent with our study design). We explored a variety of settings for these parameters to assess power as each variable changes (see Additional file 6: Table S7). Each setting tested was chosen to reflect the distribution of observed values in our real Hi-C data. For each configuration of parameters, we performed the following simulation: We simulated genotypes by randomly sampling a set of alleles (one allele per subject) from a binomial distribution parameterized by the number of subjects and the MAF; we repeated this process twice and create per-subject genotypes by adding the results of the sampling of alleles. We simulated per-subject random effects, and per-sample residuals. To obtain a given sample’s simulated Hi-C contact matrix value, we added the mean Hi-C contact matrix value to that sample’s simulated genotype (multiplied by the pre-specified effect size), the specific subject’s random intercept, and the sample’s random residual. After performing this for all samples, we then fitted the same LMM model used in our QTL search. We repeated this simulation and model fitting process 1000 times and computed power as the fraction of times the null hypothesis that the effect of genotype is equal to 0 is rejected at a nominal *p* value of 0.05.

#### FIRE, DI, and INS QTL searches

##### FIRE tested bins and SNPs

We limited our FIRE QTL search to the subset of testable bins that were called as FIRE in at least one YRI LCL (*N* = 5822 FIRE test bins), and the subset of testable SNPs therein (*N* = 128,137 FIRE test SNPs).

##### INS tested bins and SNPs

For the INS-QTL search, we examined 328,530 test SNPs with 12,976 variable INS bins (see the “*F* test for variable bins” section).

##### DI tested bins and SNPs

For the DI-QTL search, we examined 181,950 test SNPs with 7590 variable DI bins (see the “*F* test for variable bins” section). For the DI-QTL search, we further classified each DI bin based on which whether it showed stronger upstream or downstream bias, because we saw a Simpson’s paradox when we considered them together (see Additional file 1: Figure S10b). This was done as follows: for each bin, we evaluated the DI score in each of 11 unrelated YRIs and identified the DI score among these individuals with the largest absolute value. We defined a bin as “upstream DI bias” if the DI score with the highest absolute value was positive, or “downstream DI bias,” if the DI score with the highest absolute value was negative. Only 37/7590 bins (0.4%) had individuals with both positive and negative DI values.

##### LMM QTL searches

For each test SNP, we identified the 40-Kb bin it belongs to, and fitted a linear mixed effects model, using FIRE, DI (200-Kb window; see the “Directionality index” section), or INS score (200-Kb window; see the “Insulation score” section) in each biological replicate as the response variable and genotype of that testable SNP as the explanatory variable. Since two biological replicates from the same individual are dependent, we used an individual-specific random effect to specifically characterize such within-individual dependence. We used the R package “nlme” and R function “gls” to fit the linear mixed effects model. Code is available here: http://renlab.sdsc.edu/renlab_website//download/iqtl/. The quantile-quantile plots (QQ plot) showed only minor genomic inflation (median *p* value = 0.4821, lambda = 1.0864 for FIRE-QTLs; median *p* value = 0.4864, lambda = 1.0649 for upstream-biased DI-QTLs; median *p* value = 0.4828, lambda = 1.0826 for downstream-biased DI-QTLs; median *p* value = 0.4865, lambda = 1.0646 for INS-QTLs). The linear mixed effects model identified 476, 315, 315, and 1092 SNPs with false discovery rate (FDR) less than 0.20 for FIRE, upstream-biased DI, downstream-biased DI, and INS, respectively. When more than one SNP in the same bin was identified, we selected the SNP with lowest *p* value among them to be included in the final QTL sets. After this filtering, we ended up with 387 candidate FIRE-QTLs, 268 candidate upstream-biased DI-QTLs, 277 downstream-biased DI-QTLs, and 911 candidate INS-QTLs. As a control for each of these QTL searches, we randomly shuffled the score in question (i.e., FIRE, DI, or INS) among all 11 YRI individuals and performed QTL searches on this permuted data. In each of these tests, we found no SNPs associated with the permuted scores at FDR < 0.20.

#### C-QTL search

To find QTLs affecting Hi-C contact strength, we first identified 115,187 Hi-C contact matrix cells exhibiting substantial biological variability as described in the “Normalizing Hi-C contact matrices using BNBC normalization” section, and constrained our QTL search to these cells. We then intersected these contact cells with 1,304,404 testable SNPs by requiring a SNP to sit in one anchor bin of one of these variable matrix cells. We also filtered out matrix cells to ensure both anchor bins of the matric cell are among 51,511 testable bins. In total, we obtained 3,109,039 tests involving 687,655 SNPs and 54,880 matrix cells on all 22 autosomes. For each test, we used the BNBC normalized data described in the “Variable regions” section, but used only the 11 unrelated YRI individuals with genotypes available and fit a linear mixed effects model in which genotype is a fixed effect and subject is a random intercept. We then used “lmerTest” package in R to estimate *p* values for the fixed effect of genotype [70]. We used the IHW framework [50] to estimate FDR, with the distance between anchor bins as an informative covariate, and call any matrix cell with FDR < 0.2 as significant. We further filtered significant tests by selecting the most significant SNPs per matrix cell and kept the leftmost SNPs among SNPs in perfect LD in two anchor bins of the matrix cell. After filtering, we ended up with 463 tests involving 345 SNPs and 463 matrix cells. To make the aggregate contact plots in Fig. 4g, we recoded the genotypes based on the direction of effect such that 0, 1, 2 refer to the genotypes containing 0, 1, or 2 alleles associated with the increased phenotype, respectively. Next, to avoid aggregating the same submatrix multiple times, we filtered by (1) selecting only the most significant matrix cell associated with each QTL and (2) selecting only the most significant QTL associated with each anchor bin (in some cases the same bin anchors multiple matrix cells associated with different QTL SNPs). This filtering left 165 unique matrix cell QTL interactions for plotting. For each matrix cell, we then extracted a submatrix including 25 bins upstream and 25 bins downstream. Submatrices with missing values were discarded. For each QTL, we then calculated the mean submatrix values for each genotype and then subtracted submatrices to calculate the difference in interaction frequency between the 1 and 0 genotypes, and between the 1 and 2 genotypes. These differences were then averaged across QTLs and plotted in Fig. 4g.

#### Validation of QTLs in additional individuals

Our validation set included six unrelated individuals not included in the discovery set: NA12878, NA19240, HG00512, HG00513, HG00731, and HG00732. For each QTL, we collected the genotype among six additional individuals, and the corresponding FIRE, DI, or INS scores. Note that a small fraction of QTLs have missing genotypes in these six individuals (coded as “-1”), and these missing data points were eliminated from validation analysis. We examined the distributions of scores for each genotype. For each QTL type (i.e., FIRE, DI, or INS), we found that the same direction of effect observed in the discovery set is observed on average in the validation set. To assess the significance of this observation, we approximated the null expectation as follows. For FIRE-QTLs, for example, we started from all 128,137 FIRE test SNPs and 5822 FIRE test bins. Note that in our discovery set, we identified 387 FIRE-QTLs, each in a different 40-Kb bin. To create a random control SNP group, we first randomly selected 387 40-Kb bins from all 5822 FIRE test bins. Next, within each select bin, we randomly selected one SNP and combined all these 387 selected SNPs into a control SNP group. We then tested their SNP effect on the six additional individuals. We repeated such sampling with replacement 1000 times, to create a null distribution of positive and negative SNP effect, respectively. We performed the same type of permutations for DI and INS. Similar analysis was performed for C-QTLs with a few modifications. First, we only used replicates from NA19420, HG00512, HG00513, HG00731, and HG00732 as explained in the “Variable regions” section. Second, 1000 random permutations were performed by sampling matrix cells instead of bins. Third, we used values of biological replicates separately instead of as merged data because the BNBC normalization is performed at the level of replicates.

#### Examining epigenetic variation at FIRE, DI, INS, and C-QTLs

To examine epigenetic variation at 3D genome QTLs, we re-analyzed DNase-seq data from 59 LCLs [38], histone modification ChIP-Seq data (H3K27ac, H3K4me1 and H3K4me3) for 65 LCLs [39], and CTCF ChIP-seq data from 11 LCLs [55]. These data were re-mapped using the WASP pipeline to control for allelic mapping artifacts and calculating the signal in 40-Kb bins as described above in the "Alignment with WASP" section. We examined the effect of genotype at FIRE, DI, INS, or C-QTLs on DNase-seq and ChIP-seq signal by linear regression. As a control, we randomly selected the matched number of SNPs with the same approach described in the “Validation of QTLs in additional individuals” section and re-did such validation analysis. We repeated such random sampling 1000 times to create the empirical null distribution of no genetic effect. For C-QTLs, we used the sum of epigenetic features in two anchor bins to calculate correlation with contact frequency.

### Nominal fraction analyses

#### Comparing between 3D chromatin QTL types

To compare between different 3D chromatin QTLs, we took the raw test results for each QTL set and projected other 3D QTLs into the test results. For example, in Fig. 4j, we selected subset of SNPs that are DI-QTLs and plotted them (dark green dots) using *p* values from FIRE-QTLs along with all SNPs tested in the FIRE-QTL search (black dots). We also used all tested SNPs in the DI-QTL search (light green dots) as a control set. To assign significance to the overlap, we compared the fraction of SNPs with nominal significance (*p* < 0.05) in each set: (1) DI-QTL tested SNPs that were not significant QTLs and (2) DI-QTLs. We calculated *p* values for this comparison by chi-square test. To rule out the effect of sampling bias when selecting a small number of SNPs, we also performed permutation. In each permutation, we randomly selected the same number of SNPs as the real QTL set (from the full set of tested SNPs) and calculated the fraction with nominal significance. We then computed bootstrap *p* values using 10,000 such permutations under the null hypothesis that the fraction of nominal significance is the same between QTLs and random selected SNPs. For C-QTLs, one SNP may be tested against multiple matrix cells, so we only kept the most significant *p* value for each SNP to avoid biases toward SNPs with multiple tests.

#### Comparing 3D chromatin QTLs to other molQTLs

Similar approaches were used to assess overlap between 3D chromatin QTLs and other molQTLs. We obtained full test results (all tested SNPs with the *p* values) from previous molQTL studies and projected 3D chromatin QTLs into those test results. We calculated the fraction of nominal significance and used chi-square test to evaluate significance between 3D-QTLs and non-3D-QTLs. Similarly, we performed bootstrap to estimate significance empirically. One modification is that we extended our QTL sets by incorporating all SNPs in perfect LD with the same 40-Kb bin because we may not use the same tagging SNP in our study as used in other studies. To ensure a fair comparison, we performed the same extension for the control sets of all tested SNPs.

#### Comparing 3D chromatin QTLs to GWAS

Comparison with the GWAS results was performed in the same manner as described in the “Comparing 3D chromatin QTLs to other molQTLs” section for other molQTLs. Instead of test results for other molQTLs, we used summary statistics from previous GWAS.

### FISH

#### Cell preparation for FISH

Approximately 100,000 cells were adhered to center of PDL-coated coverslips (Neuvitro, GG-22-15-PDL) by placing 100 uL of cells at 1 × 10 [6] cells/mL. Cells on coverslips were incubated for an hour at 37 °C, carefully washed with PBS, and fixed with 4% paraformaldehyde in 1× PBS for 10 min. PFA was quenched with 0.1 M Tris-Cl, pH 7.4 for 10 min, washed with PBS, and stored in 1× PBS at 4 °C for up to 1 month.

#### BAC probe labeling and preparation

All BAC clones were ordered from the BACPAC Resource Center at the Children’s Hospital Oakland Research Institute: “U” probe is RP11-74P5, “C” probe is RP11-337 N12, and “D” probe is RP11-248 M23. BAC DNAs were labeled with either Chromatide Alexa Fluor 488-5 dUTP (Invitrogen, C-11397) or Alexa Fluor 647-aha-dUTP (Invitrogen, A32764) using nick-translation kit (Roche, 10976776001), and incubated in 15 °C for 4 h. The nick-translation reaction was deactivated using 1 μL of 0.5 M EDTA, pH 8.0, and heated for 10 min at 65 °C. The probes were then purified using illustra ProbeQuant G-50 Micro Columns (GE Healthcare, 28903408) and eluted to a concentration of 20 ng/μL. Probes were mixed with Human Cot-1 DNA (Invitrogen, 15279011) and salmon sperm (Invitrogen, 15632011), and precipitated with 1/10th volume of 3 M sodium acetate, pH 5.2, and 2.5 volume of absolute ethanol for at least 2 h at − 20 °C. Probes were then spun down, washed with cold 70% ethanol, resuspended in formamide and 40% dextran sulfate in 8X SSC, and incubated at 55 °C.

#### Hybridization

Cells on coverslips were blocked with 5% BSA and 0.1% Triton X-100 in PBS for 30 min at 37 °C and washed twice with 0.1% Triton X-100 in PBS for 10 min each with gentle agitation at room temperature. Cells were permeabilized with 0.1% saponin and 0.1% Triton X-100 in PBS for 10 min at room temperature. Next, they were incubated in 20% glycerol in PBS for 20 min, freeze-thawed three times with liquid nitrogen, and incubated in 0.1 M hydrogen chloride at room temperature for 30 min. Cells were further blocked for 1 h at 37 °C in 3% BSA and 100 μg/mL RNase A in PBS. Cells were permeabilized again with 0.5% saponin and 0.5% Triton X-100 in PBS for 30 min at room temperature. Lastly, they were rinsed with 1× PBS and washed with 2× SSC for 5 min. For hybridization of probes, the prepared probes were denatured at 73 °C for 5 min in water bath. Cells were denatured in a two-step process in a 73 °C water bath: 2.5 min in 70% formamide in 2× SSC and 1 min in 50% formamide in 2× SSC. Denatured probes were transferred onto microscope slides, and coverslips were placed on top with cell-side facing down. The coverslips were sealed with rubber cement and incubated overnight at 37 °C in a dark, humid chamber. Next day, coverslips were carefully removed and transferred onto a 6-well plate. Cells were washed at 37 °C with gentle agitation, twice with 50% formamide in 2× SSC for 15 min and three times with 2× SSC for 5 min. The cells were then stained with DAPI (Invitrogen, D1306), mounted on microscope slides with ProLong Gold Antifade Mountant (Invitrogen, P36930), sealed with nail polish, and imaged.

#### Microscope and analysis

Images were acquired with DeltaVision RT Deconvolution Microscope at UC San Diego’s department of neuroscience (acquired with award NS047101). Captured images were processed using the TANGO [71] plugin in ImageJ for quantitative analysis. Each FISH experiment contained two probes labeled with different color dyes (either U-C or C-D). We limited our analysis to nuclei containing 2 labeled foci for each color (4 total foci), allowing us to more confidently distinguish foci *in cis* from those *in trans*. Distances were measured from the center of one color focus to the center of the closest focus of the other color.

### Re-analysis of public datasets

#### Analysis of ChIP-seq data from Kasowski et al. and McVicker et al.

Raw fastq files were downloaded from SRA database for each experiment (SRP030041 and SRP026077, respectively). Reads were aligned to hg19 reference genome using BWA MEM (Kasowski) or BWA ALN [60] v0.7.8 (McVicker) with WASP pipeline [40, 45] to eliminate allelic mapping bias. Only reads with high mapping quality (> 10) were kept. PCR duplicates were removed using Picard tools v1.131 (http://broadinstitute.github.io/picard). MACS2 [72] v2.2.1 was then used to call peaks using corresponding input files. For CTCF and SA1, default parameters were used for MACS2. For H3K27ac, H3K4me1, and H3K4me3, peak calling was done using “--nomodel” parameter because we do not expect sharp peaks for histone modifications. For H3K27me3 and H3K36me3, peak calling was done using “--nomodel --broad” parameter. Bigwig files were generated by MACS2 using fold enrichment for viewing in genome browser. All Kasowski data were processed in pair-end mode, and both replicates were merged for analysis. All McVicker data were processed in single-end mode, and the pooled input data were used for all samples because there are no individual input files. To compute signals in peaks, we used a set of merged peaks across all individuals for each mark.

#### Analysis of RNA-seq data from Kasowski et al.

Raw fastq files were downloaded from SRA database (SRP030041). Reads were aligned to hg19 reference genome using STAR [73] v2.4.2a with the WASP pipeline in pair-end mode to eliminate allelic mapping bias. Gencode [74] v24 annotation was used to construct STAR index and computing FPKM. Only uniquely mapped reads were kept. Cufflinks [75] v2.2.1 was applied to compute FPKM values. Both replicates were merged for analysis.

#### Analysis of DNase-seq data from Degner et al.

Raw fastq files were downloaded from SRA database for each experiment (SRP007821). Reads were aligned to hg19 reference genome using BWA ALN with the WASP pipeline in single-end mode to eliminate allelic mapping bias. Only reads with high mapping quality (> 10) were kept. PCR duplicates were removed using Picard tools. Bigwig files were generated using makeUCSCfile commands in homer tools [76] v4.9.1.

#### Analysis of ChIP-seq data from Ding et al.

Raw fastq files were downloaded from SRA database for each experiment (SRP004714). Reads were aligned to hg19 reference genome using BWA MEM v0.7.8 with the WASP pipeline to eliminate allelic mapping bias. Only reads with high mapping quality (> 10) were kept. PCR duplicates were removed using Picard tools. We performed quality control for CTCF ChIP-seq data by FRIP (Fraction of Reads In Peaks) and used datasets with FRIP > 10. Bigwig files were generated using bamCoverage commands in deepTools [77] v2.3.3. To compute signals in peaks, we used the merged CTCF peaks from Kasowski data.

#### Analysis of ChIP-seq data from Grubert et al.

Bigwig files and peaks for H3K27ac, H3K4me1, and H3K4me3 were downloaded from GEO database (GSE62742). Peaks for each mark were merged and then used to compute the averaged signal.

## Supplementary information

**Additional file 1: Figure S1.** Hi-C derived molecular phenotypes measured across 20 LCLs. **FigureS2.** FIRE measures density of local interactions. **Figure S3.** Aggregate looping interactions in each sample. **Figure S4.** 3D chromatin variation among 20 LCLs and H1-derived lineages. **Figure S5.** Characterization of variable regions of 3D chromatin conformation. **Figure S6.** Additional characterization of variable regions of 3D chromatin conformation. **FigureS7.** Coordinated variation between 3D chromatin conformation and multiple molecular phenotypes. **Figure S8.** Correlations between 3D genome phenotypes and other molecular phenotypes. **Figure S9.** 3D chromatin QTLs. **Figure S10.** Influence of 3D chromatin QTLs on epigenomic and disease phenotypes.

**Additional file 2: Tables S1-S3.** Public datasets re-analyzed in this study, LCLs included in the study, and Hi-C mapping statistics.

**Additional file 3: Table S4.** TAD and boundary calls for each sample.

**Additional file 4: Table S5.** Regions showing evidence of biological variability in 3D chromatin conformation.

**Additional file 5: Table S6.** Matrix cells showing evidence of biological variability.

**Additional file 6: Table S7.** Summary of phasing results.

**Additional file 7: Table S8.** Power calculations at standard *p <* 0.05 and at *p-*values corresponding to FDR < 0.2 (FIRE-QTLs *p <* 7e-4, INS-QTL *p <* 7e-4, DI-QTL *p <* 7e-4, C-QTL *p <* 3e-5).

**Additional file 8: Table S9.** 3D chromatin conformation QTLs.

**Additional file 9: Table S10.** Overlaps between 3D chromatin QTL and GWAS catalog.

**Additional file 10.** Review history.

## Data Availability

All raw sequencing data and many processed data files are available through NCBI’s Gene Expression Omnibus (GEO) accession GSE128678 [78], as well as through the 4D Nucleome data portal (https://data.4dnucleome.org). Additional processed data are available on the Ren lab’s website at http://renlab.sdsc.edu/renlab_website/download/iqtl/, or by request. The following third-party datasets were also used in this study (also listed in Additional file 2: Table S2): GSE48592 [79], GSE52457 [80], GSE63525 [81], GSE50893 [82], GSE47991 [83], GSE62742 [84], E-ERAD-141 [85], and GSE31388 [86]. Code is available at Zenodo accession 3475719 [87], and released under a Common Development and Distribution License compliant with OSI (http://opensource.org/licenses).
