# Supplementary material for: Common DNA sequence variation influences 3-dimensional conformation of the human genome
Source: Genome Biol. 2019 Nov 28;20:255. doi: 10.1186/s13059-019-1855-4 (PMC6883528; doi:10.1186/s13059-019-1855-4)
Supplement: Supplementary file 1 — Additional file 1: Figure S1. Hi-C derived molecular phenotypes measured across 20 LCLs. FigureS2. FIRE measures density of local interactions. Figure S3. Aggregate looping interactions in each sample. Figure S4. 3D chromatin variation among 20 LCLs and H1-derived lineages. Figure S5. Characterization of variable regions of 3D chromatin conformation. Figure S6. Additional characterization of variable regions of 3D chromatin conformation. FigureS7. Coordinated variation between 3D chromatin conformation and multiple molecular phenotypes. Figure S8. Correlations between 3D genome phenotypes and other molecular phenotypes. Figure S9. 3D chromatin QTLs. Figure S10. Influence of 3D chromatin QTLs on epigenomic and disease phenotypes. [file 13059_2019_1855_MOESM1_ESM.pdf]

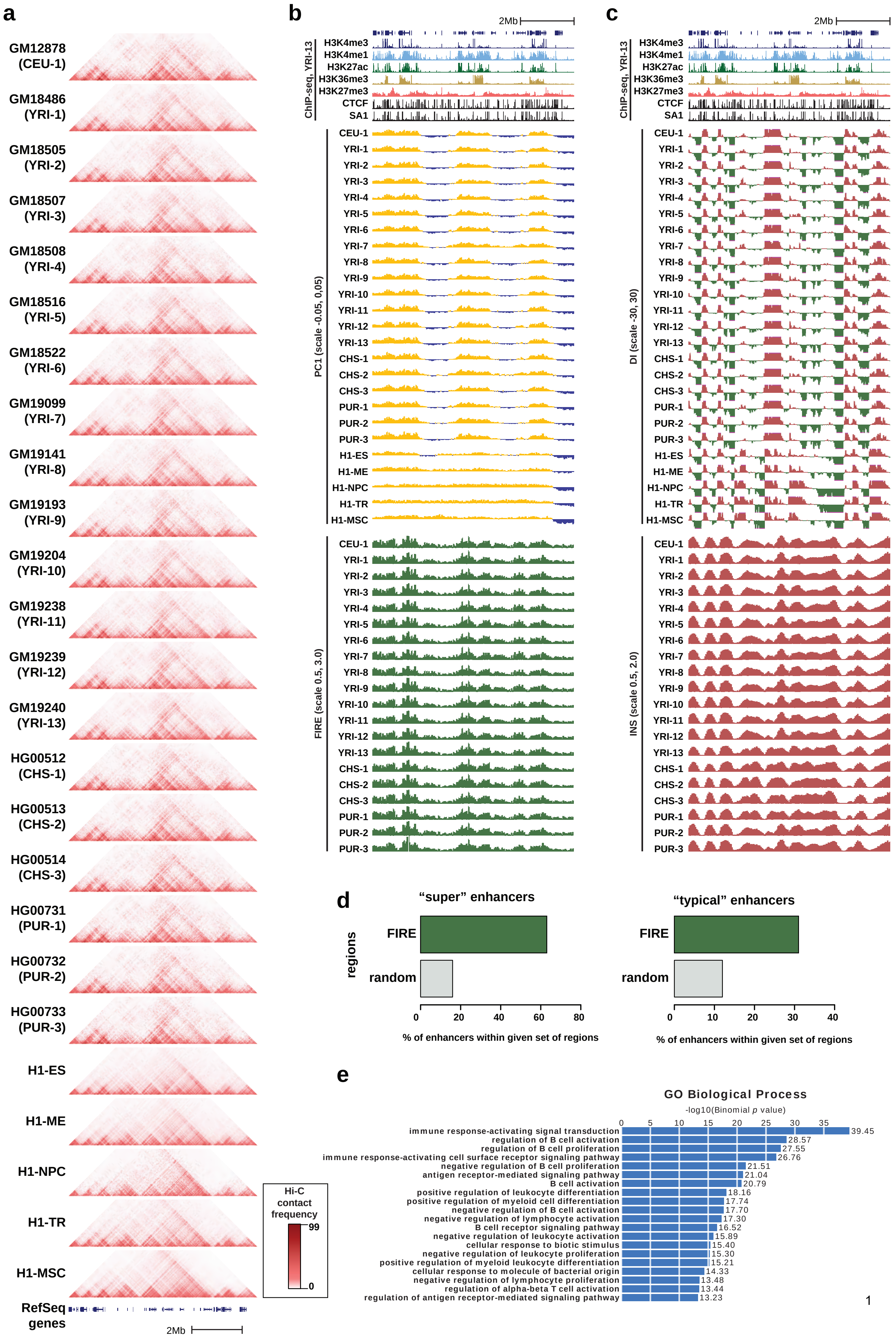

a
GM12878 (CEU-1)
GM18486 (YRI-1)
GM18505 (YRI-2)
GM18507 (YRI-3)
GM18508 (YRI-4)
GM18516 (YRI-5)
GM18522 (YRI-6)
GM19099 (YRI-7)
GM19141 (YRI-8)
GM19193 (YRI-9)
GM19204 (YRI-10)
GM19238 (YRI-11)
GM19239 (YRI-12)
GM19240 (YRI-13)
HG00512 (CHS-1)
HG00513 (CHS-2)
HG00514 (CHS-3)
HG00731 (PUR-1)
HG00732 (PUR-2)
HG00733 (PUR-3)
H1-ES
H1-ME
H1-NPC
H1-TR
H1-MSC
RefSeq genes
2Mb
Hi-C contact frequency
99
0
b
2Mb
ChIP-seq, YRI-13
H3K4me3
H3K4me1
H3K27ac
H3K36me3
H3K27me3
CTCF
SA1
PC1 (scale -0.05, 0,05)
CEU-1
YRI-1
YRI-2
YRI-3
YRI-4
YRI-5
YRI-6
YRI-7
YRI-8
YRI-9
YRI-10
YRI-11
YRI-12
YRI-13
CHS-1
CHS-2
CHS-3
PUR-1
PUR-2
PUR-3
H1-ES
H1-ME
H1-NPC
H1-TR
H1-MSC
FIRE (scale 0.5, 3.0)
c
2Mb
DI (scale -30, 30)
INS (scale 0.5, 2.0)
d
"super" enhancers
"typical" enhancers
regions
FIRE
random
0
20
40
60
80
10
30
% of enhancers within given set of regions
e
GO Biological Process
-log10(Binomial p value)
immune response-activating signal transduction 39.45
regulation of B cell activation 28.57
regulation of B cell proliferation 27.55
immune response-activating cell surface receptor signaling pathway 26.76
negative regulation of B cell proliferation 21.51
antigen receptor-mediated signaling pathway 21.04
B cell activation 20.79
positive regulation of leukocyte differentiation 18.16
positive regulation of myeloid cell differentiation 17.74
negative regulation of B cell activation 17.70
negative regulation of lymphocyte activation 17.30
B cell receptor signaling pathway 16.52
negative regulation of leukocyte activation 15.89
cellular response to biotic stimulus 15.40
negative regulation of leukocyte proliferation 15.30
positive regulation of myeloid leukocyte differentiation 15.21
cellular response to molecule of bacterial origin 14.33
negative regulation of lymphocyte proliferation 13.48
regulation of alpha-beta T cell activation 13.44
regulation of antigen receptor-mediated signaling pathway 13.23

Figure S1. Hi-C derived molecular phenotypes measured across 20 LCLs. (a) Hi-C contact matrices show for all 20 LCLs. For comparison, we also show data from H1 human embryonic stem cells (H1-ES), and 4 lineages derived from H1 by *in vitro* differentiation[1] (H1-ME = Mesendodermal cells, H1-NPC = Neuronal precursor cells, H1-TR = Trophoblast-like cell, MSC = Mesenchymal stem cells). These H1-derived cell lines represent different cell types from the same individual (i.e. same genetic background). The region shown here is the same as Figure 1a (chr8:125,040,000-132,560,000; hg19). (b) Same region as in (a) but showing PC1 and FIRE values. ChIP-seq data for several histone modifications, CTCF, and Cohesin subunit SA1 are shown for one LCL (YRI-13, GM19240) as a reference for the epigenomic landscape[2]. (c) Same region as (a) but showing DI and INS values. (d) Bar plots show the percentage of super-enhancers (left) or typical enhancers (right) in GM12878[3] that overlap with 6,980 LCL FIRE bins (called as FIRE in at least one individual in our dataset) or 6,980 random 40kb bins. (e) Biological Process Gene Ontology terms associated with genes proximal to FIRE regions as defined by GREAT[4].

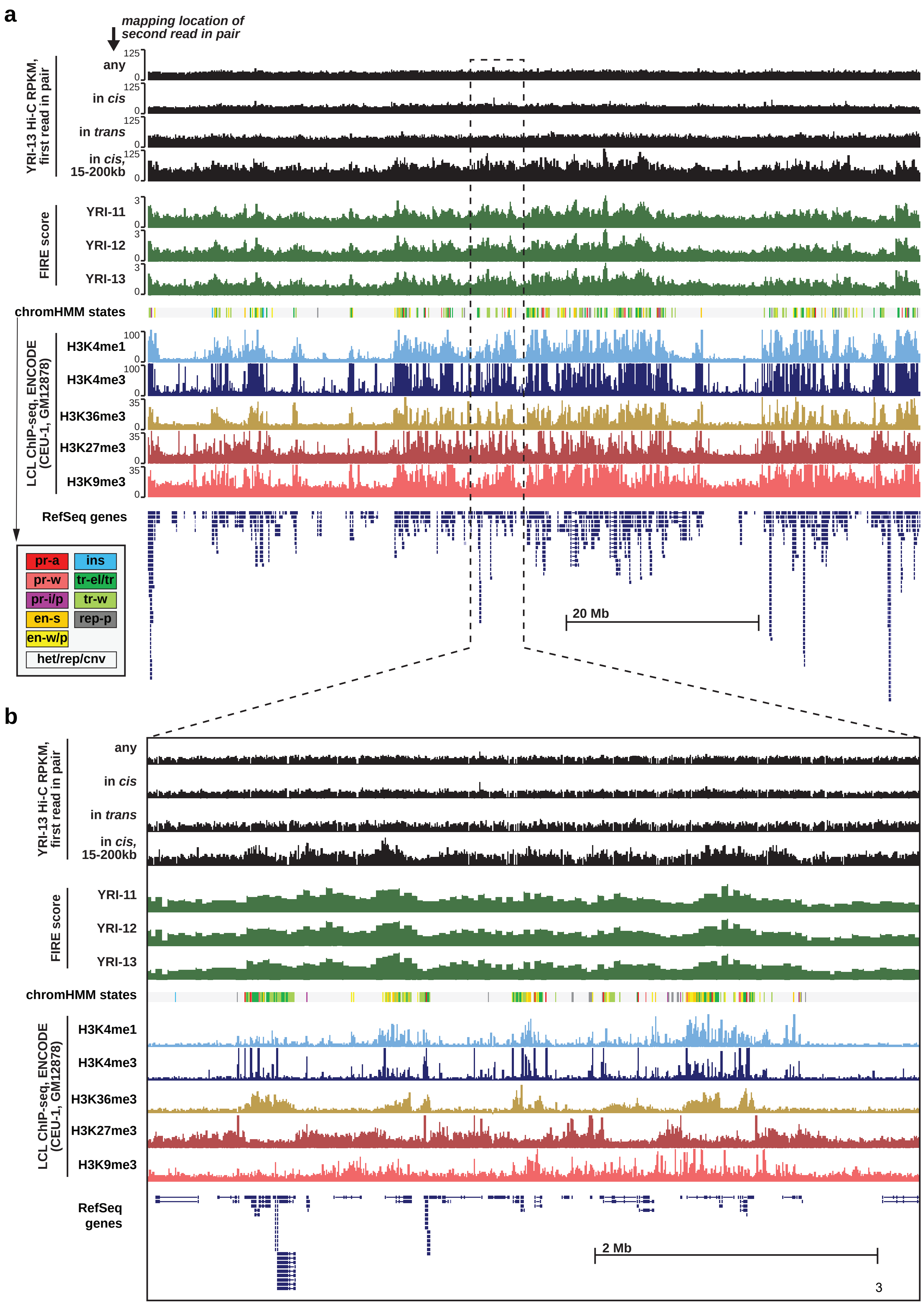
a
mapping location of second read in pair
YRI-13 Hi-C RPKM, first read in pair
any
in cis
in trans
in cis, 15-200kb
125
0
FIRE score
YRI-11
YRI-12
YRI-13
3
0
chromHMM states
LCL ChIP-seq, ENCODE (CEU-1, GM12878)
H3K4me1
H3K4me3
H3K36me3
H3K27me3
H3K9me3
100
35
RefSeq genes
pr-a
ins
pr-w
tr-el/tr
pr-i/p
tr-w
en-s
rep-p
en-w/p
het/rep/cnv
20 Mb
b
2 Mb

Figure S2. FIRE measures density of local interactions. Illustrative example showing that overall density of Hi-C reads (any read irrespective of location of interacting partner, all *cis* interactions, or all *trans* interactions) is highly consistent across the genome. However, interactions between partners separated by 15-200kb (“FIRE” distance) are enriched in regions of the genome with marks of regulatory and transcriptional activity (H3K4me1, H3K4me3, H3K36me3 from ENCODE for CEU-1 / GM12878 shown for reference)[5]. ChromHMM[6] functional annotations for CEU-1 / GM12878 are also shown. pr-a = active promoter, pr-w = weak promoter, pr-i/p = inactive/poised promoter, en-s = strong enhancer, en-w/p = weak/poised, ins = insulator, tr-el/tr = transcriptional elongation or transcriptional transition, tr-w = weak transcribed, rep-p = polycomb-repressed, het/rep/cnv = heterochromatin, low signal, repetitive, or copy number variation. Top panel (a) shows the long arm of chr14 (chr14:24,406,737-104,693,368; hg19). Bottom panel (b) is a zoomed-in view of the region boxed by dotted lines (chr14:58,000,000-63,500,000; hg19).

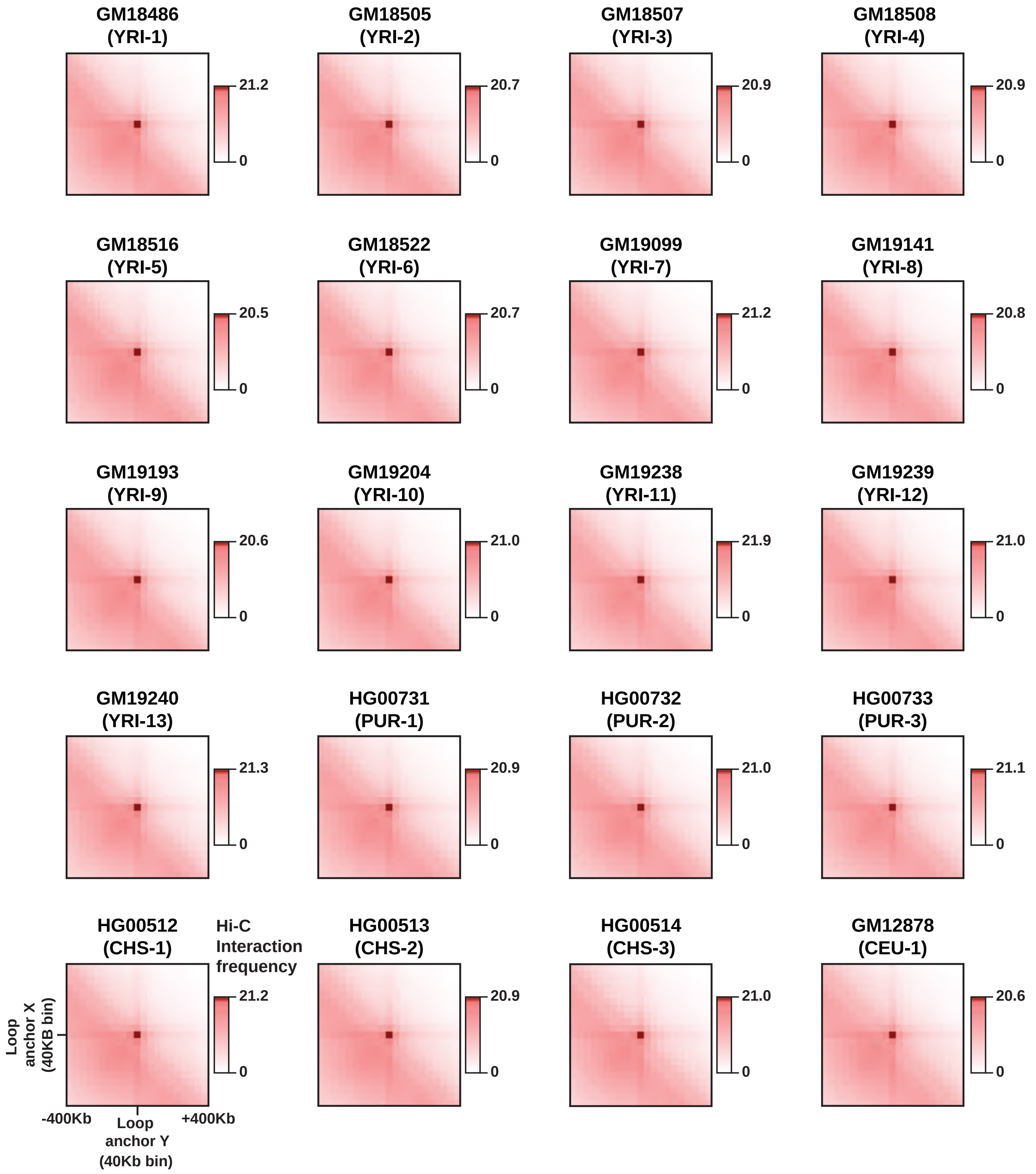

Figure S3. Aggregate looping interactions in each sample. Aggregate plots show the interaction frequencies at GM12878 HiCCUPS loops from Rao et al 2014 in each sample examined here. All autosomal *cis* loops with anchor bins separated by more than 40kb are included here (N=8,893). The middle bin represents the interaction frequency between two 40kb bins containing loop anchor bins. The full submatrix extends 10 bins (400kb) upstream and downstream of the interaction bin (x and y axis). The color scale indicates the average interaction frequency per loop from Hi-C contact matrices.

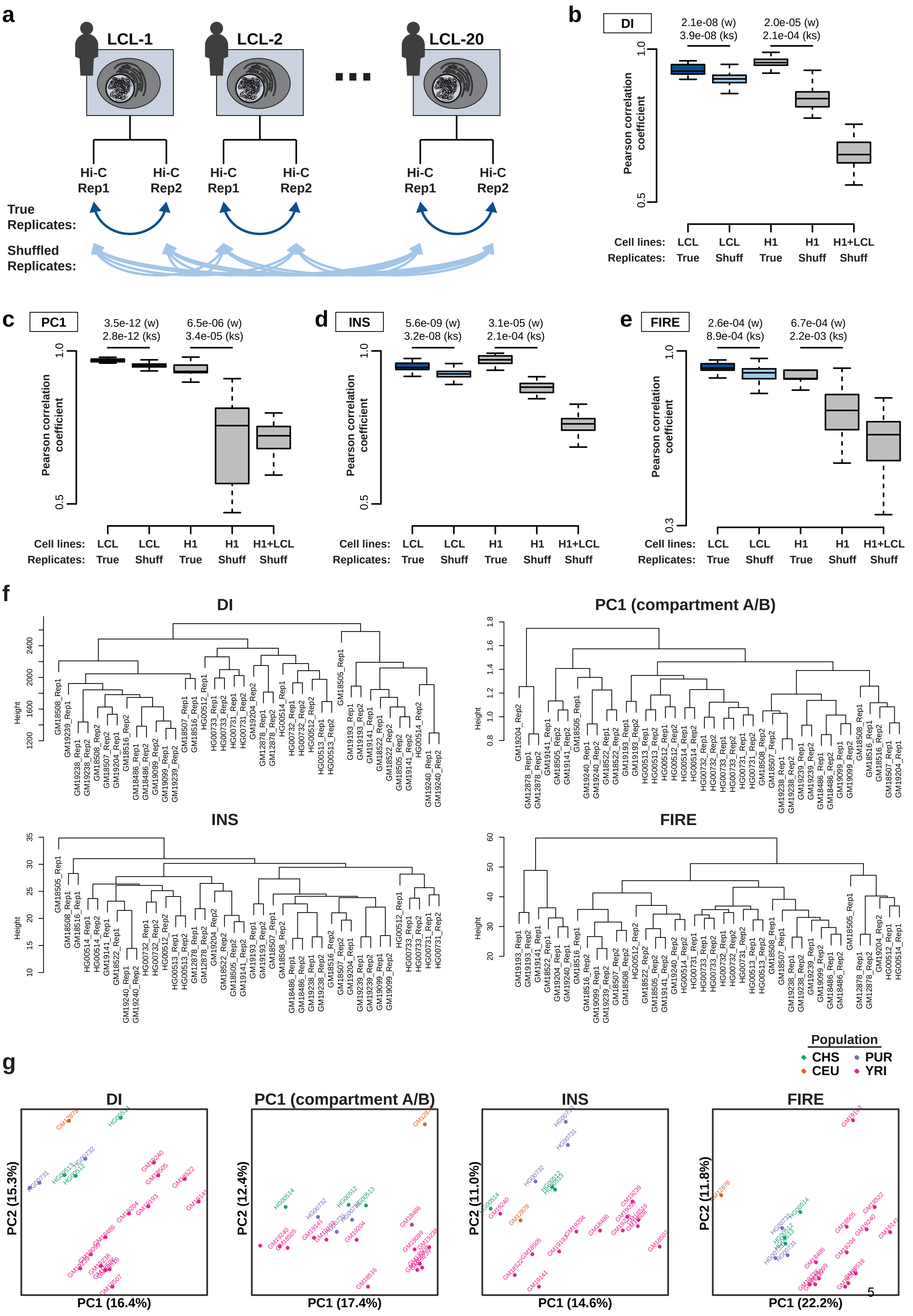
a
LCL-1
LCL-2
LCL-20
Hi-C Rep1
Hi-C Rep2
True Replicates:
Shuffled Replicates:
b
DI
2.1e-08 (w)
3.9e-08 (ks)
2.0e-05 (w)
2.1e-04 (ks)
Pearson correlation coefficient
Cell lines: LCL LCL H1 H1 H1+LCL
Replicates: True Shuff True Shuff Shuff
c
PC1
3.5e-12 (w)
2.8e-12 (ks)
6.5e-06 (w)
3.4e-05 (ks)
d
INS
5.6e-09 (w)
3.2e-08 (ks)
3.1e-05 (w)
2.1e-04 (ks)
e
FIRE
2.6e-04 (w)
8.9e-04 (ks)
6.7e-04 (w)
2.2e-03 (ks)
f
DI
PC1 (compartment A/B)
INS
FIRE
Height
g
Population
CHS
PUR
CEU
YRI
DI
PC1 (compartment A/B)
INS
FIRE
PC2 (15.3%)
PC1 (16.4%)
PC2 (12.4%)
PC1 (17.4%)
PC2 (11.0%)
PC1 (14.6%)
PC2 (11.8%)
PC1 (22.2%)

Figure S4. 3D chromatin variation among 20 LCLs and H1-derived lineages.
(a) Shuffling scheme used to assess biological variability in Figure 1b-d, and here in panels b-e. (b)-(e) Boxplots show Pearson correlation coefficient between biological replicates from the same cell line (Replicates = “True”), and between replicates from different cell lines (Replicates = “Shuff”; short for “shuffled”). The set of cell lines considered is indicated below each box. LCL = 20 LCL cell lines (40 replicates); H1 = H1-ES and the four derived lineages H1-ME, H1-NPC, H1-TB, H1-MSC (5 cell lines, 10 replicates); H1+LCL = 20 LCLs and 5 H1-derived lines considered together (50 replicates). All phenotypes examined here (DI, PC1, INS, or FIRE) show a signature of cell-type specificity whereby they are more similar across individuals when looking at the same cell type (i.e. LCLs), relative to comparing across cell types within an individual (i.e. same genetic background, H1s). Statistical significance calculated by two-sided Wilcoxon rank sum test (labeled w), or Kolmogorov–Smirnov test (labeled ks). (f) Dendrograms from hierarchical clustering of 40 Hi-C replicates based on the Hi-C-derived phenotype indicated above each dendrogram (DI, PC1, INS, or FIRE). In most cases, replicates from the same cell lines cluster together. (g) Principal Component Analysis of 20 LCLs using one of four Hi-C-derived phenotypes, as indicated above each plot. Each population in our study is represented by a different color as indicated in the color key to the right.

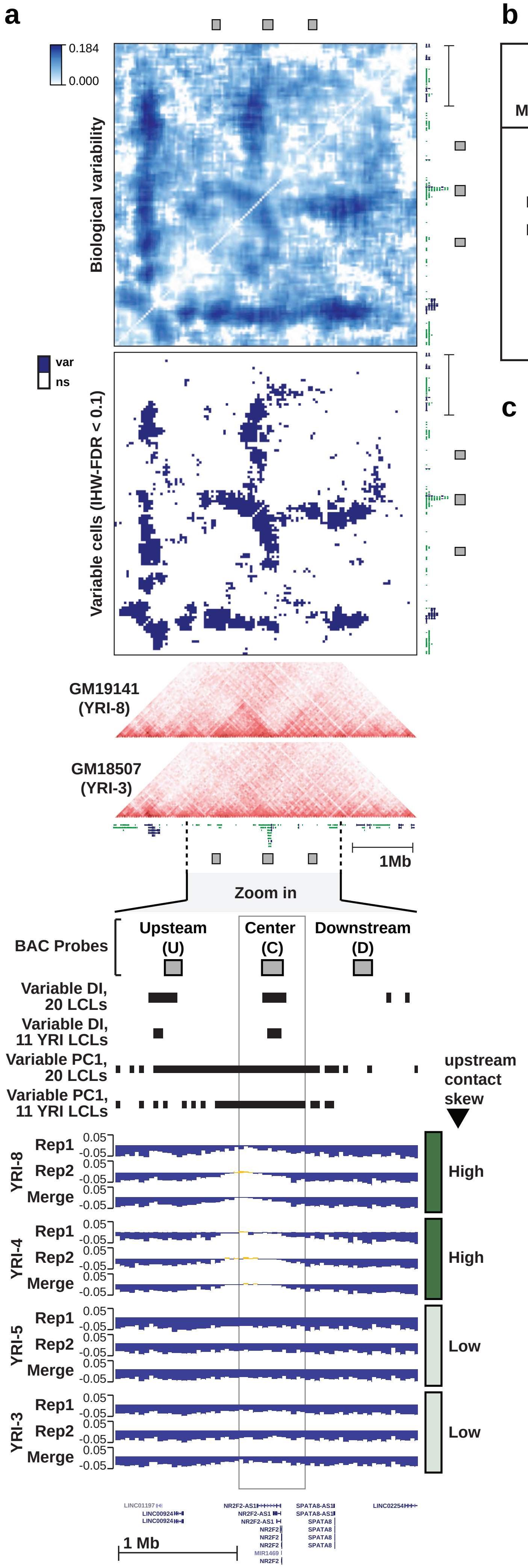

b

Metric	discovery set	testable bins	variable bins (limma FDR<0.1)	merged regions	empirical false positives (avg 1K permutations)	empirical FDR
DI	20 LCLs	17,178	3,424	2,318	59.6	5.49%
DI	11 YRI LCLs	16,605	1,086	818	13.6	0.40%
FIRE	20 LCLs	7,276	3,219	1,993	29.2	8.92%
FIRE	11 YRI LCLs	6,321	327	255	24.3	0.75%
INS	20 LCLs	15,387	6,661	2,485	47.3	1.93%
INS	11 YRI LCLs	14,319	2,447	1,033	7.9	0.12%
PC1	20 LCLs	52,328	19,128	7,732	150.6	2.22%
PC1	11 YRI LCLs	52,430	6,777	3,360	24.2	0.13%

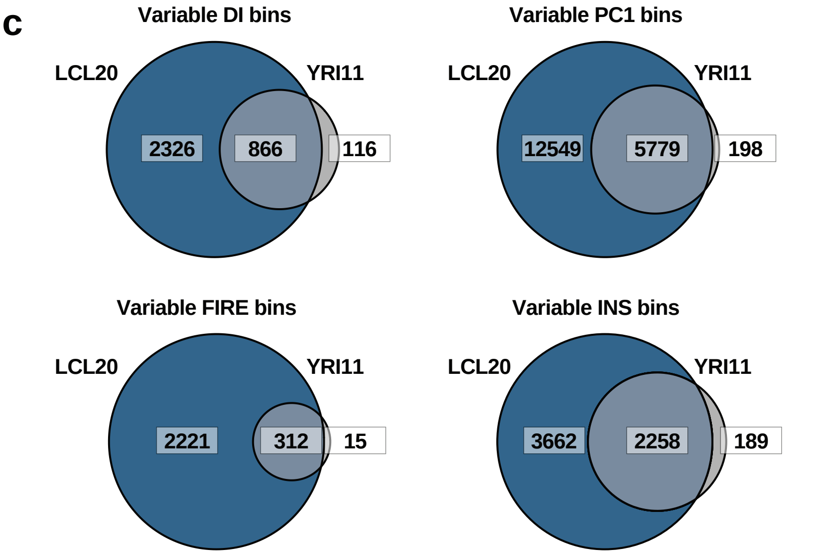

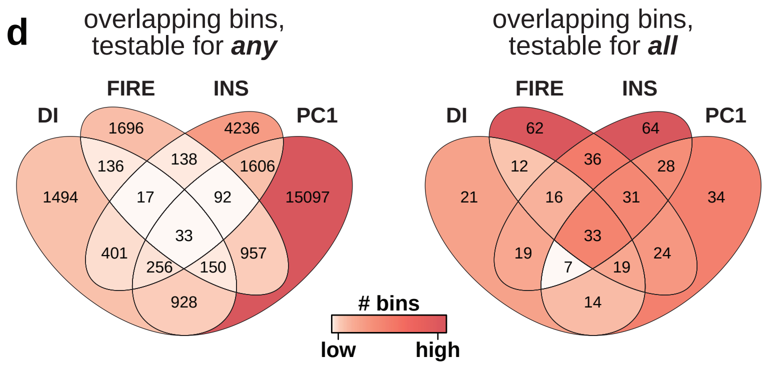

Figure S5. Characterization of variable regions of 3D chromatin conformation. (a) Same region as in Figure 2a (chr15:94,280,000-99,280,000), but showing reproducible variation in PC1, and full square matrices for contact matrix variability as opposed to the half-matrices shown in 2a. FISH probes used in Figure 2 are represented as grey boxes to the top and right of square heatmaps. (b) Similar to Figure 2b, but with additional data columns. The "merged regions" column shows the number of regions after merging variable bins that are immediately adjacent to each other. The empirical false positives and FDR columns show the number and percentage of false positive variable regions detected in 1,000 permutations with shuffled labels. (c) Venn diagrams showing the overlap of variable regions identified using either all 20 LCLs ("LCL20") or only the 11 unrelated YRI LCLs ("YRI11"). Only bins tested for both LCL20 and YRI11 are included here. (d) Venn diagrams showing the number of variable bins for each phenotype or combination of phenotypes. The diagram on the left includes bins that were tested for at least one of the four phenotypes. The diagram on the right only includes bins that were tested for all four phenotypes.

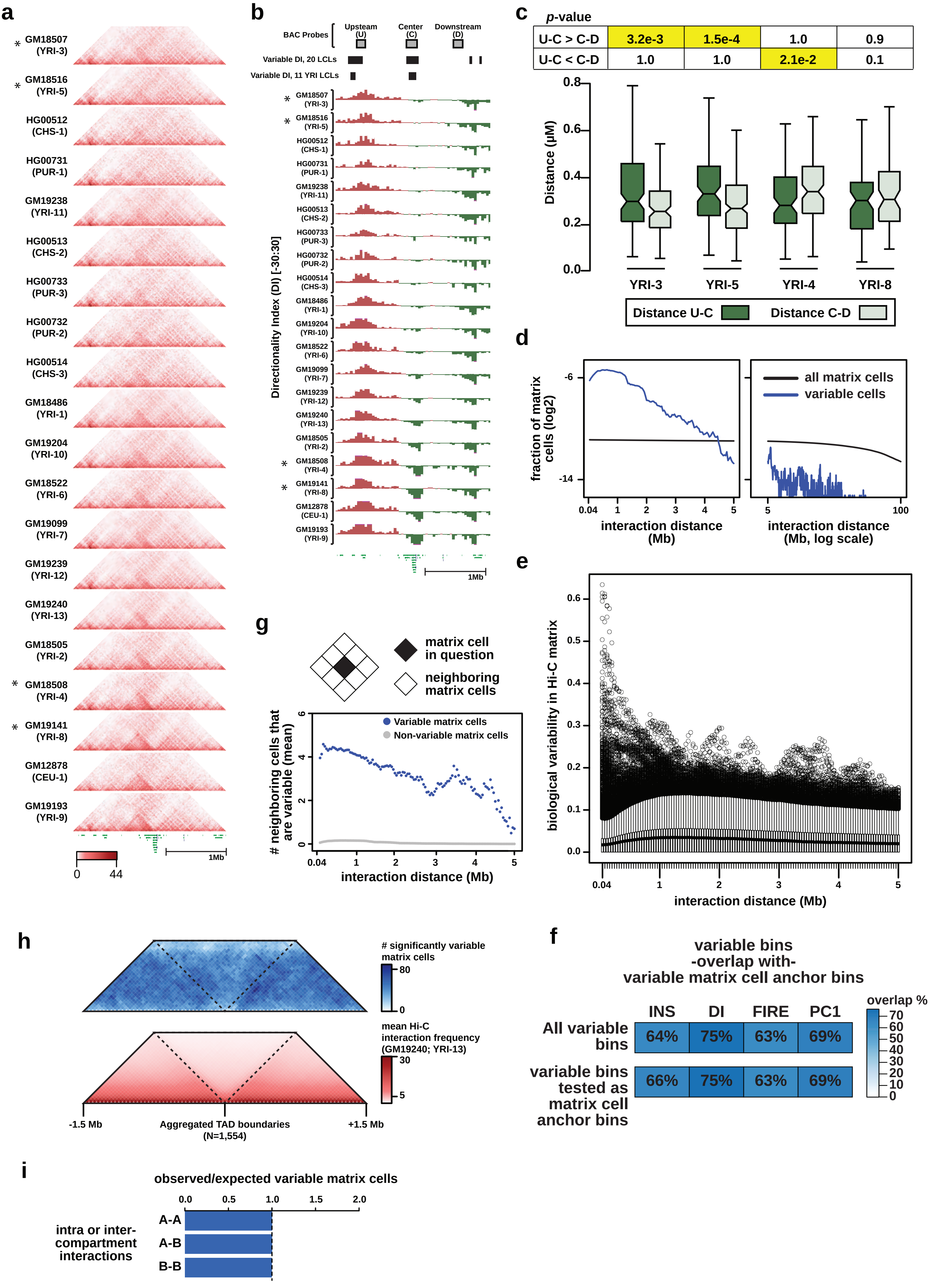
a
GM18507 (YRI-3)
GM18516 (YRI-5)
HG00512 (CHS-1)
HG00731 (PUR-1)
GM19238 (YRI-11)
HG00513 (CHS-2)
HG00733 (PUR-3)
HG00732 (PUR-2)
HG00514 (CHS-3)
GM18486 (YRI-1)
GM19204 (YRI-10)
GM18522 (YRI-6)
GM19099 (YRI-7)
GM19239 (YRI-12)
GM19240 (YRI-13)
GM18505 (YRI-2)
GM18508 (YRI-4)
GM19141 (YRI-8)
GM12878 (CEU-1)
GM19193 (YRI-9)
1Mb
0
44
b
BAC Probes
Upstream (U)
Center (C)
Downstream (D)
Variable DI, 20 LCLs
Variable DI, 11 YRI LCLs
Directionality Index (DI) [-30:30]
1Mb
c
p-value
U-C > C-D 3.2e-3 1.5e-4 1.0 0.9
U-C < C-D 1.0 1.0 2.1e-2 0.1
Distance (μM)
YRI-3
YRI-5
YRI-4
YRI-8
Distance U-C
Distance C-D
d
fraction of matrix cells (log2)
interaction distance (Mb)
interaction distance (Mb, log scale)
all matrix cells
variable cells
e
biological variability in Hi-C matrix
interaction distance (Mb)
g
matrix cell in question
neighboring matrix cells
neighboring cells that are variable (mean)
Variable matrix cells
Non-variable matrix cells
interaction distance (Mb)
h
significantly variable matrix cells
mean Hi-C interaction frequency (GM19240; YRI-13)
-1.5 Mb
Aggregated TAD boundaries (N=1,554)
+1.5 Mb
f
variable bins -overlap with- variable matrix cell anchor bins
INS DI FIRE PC1
All variable bins 64% 75% 63% 69%
variable bins tested as matrix cell anchor bins 66% 75% 63% 69%
overlap %
i
observed/expected variable matrix cells
intra or inter-compartment interactions
A-A
A-B
B-B

Figure S6. Additional characterization of variable regions of 3D chromatin conformation. (a-b) Same region as Figure 2a but showing Hi-C data (a) or DI (b) for all 20 LCLs ordered by the degree of upstream contact bias in the middle region. (c) Same underlying FISH data as in Figure 2e, but here comparing the distance between U and C probes to the distance between C and D probes within the same LCL. *p*-values calculated by one-sided Wilcoxon rank sum test. (d) As in Figure 2d, blue line shows the fraction of variable matrix cells distributed across a range of interaction distances. Black line shows the fraction of all matrix cells distributed across the same range of interaction distances. (e) Boxplots show average biological variability for matrix cells separated by distances as indicated on the X axis (40Kb – 5Mb in 40Kb increments). Outliers show as open circles above boxplots. (f) The fraction of variable INS/DI/FIRE/PC1 bins that overlap variable matrix cell anchor bins. (g) Clustering of variable matrix cells as a function of contact distance (40Kb – 5Mb in 40Kb increments). Blue dots show values for variable matrix cells. Grey dots show values for non-variable matrix cells (i.e. matrix cells in which we did not detect significant biological variability among our samples). (h) Bottom triangle shows the average Hi-C contact frequency at LCL TAD boundaries (from GM12878) and adjacent regions. Top triangle plot shows the number of variable matrix cells identified at the indicated positions relative to these TAD boundaries. (i) The ratio of observed to expected variable matrix cells among contacts between regions the same compartment (A-A or B-B) or in different compartments (A-B). Expected values were derived from the total number of matrix cells in each category.

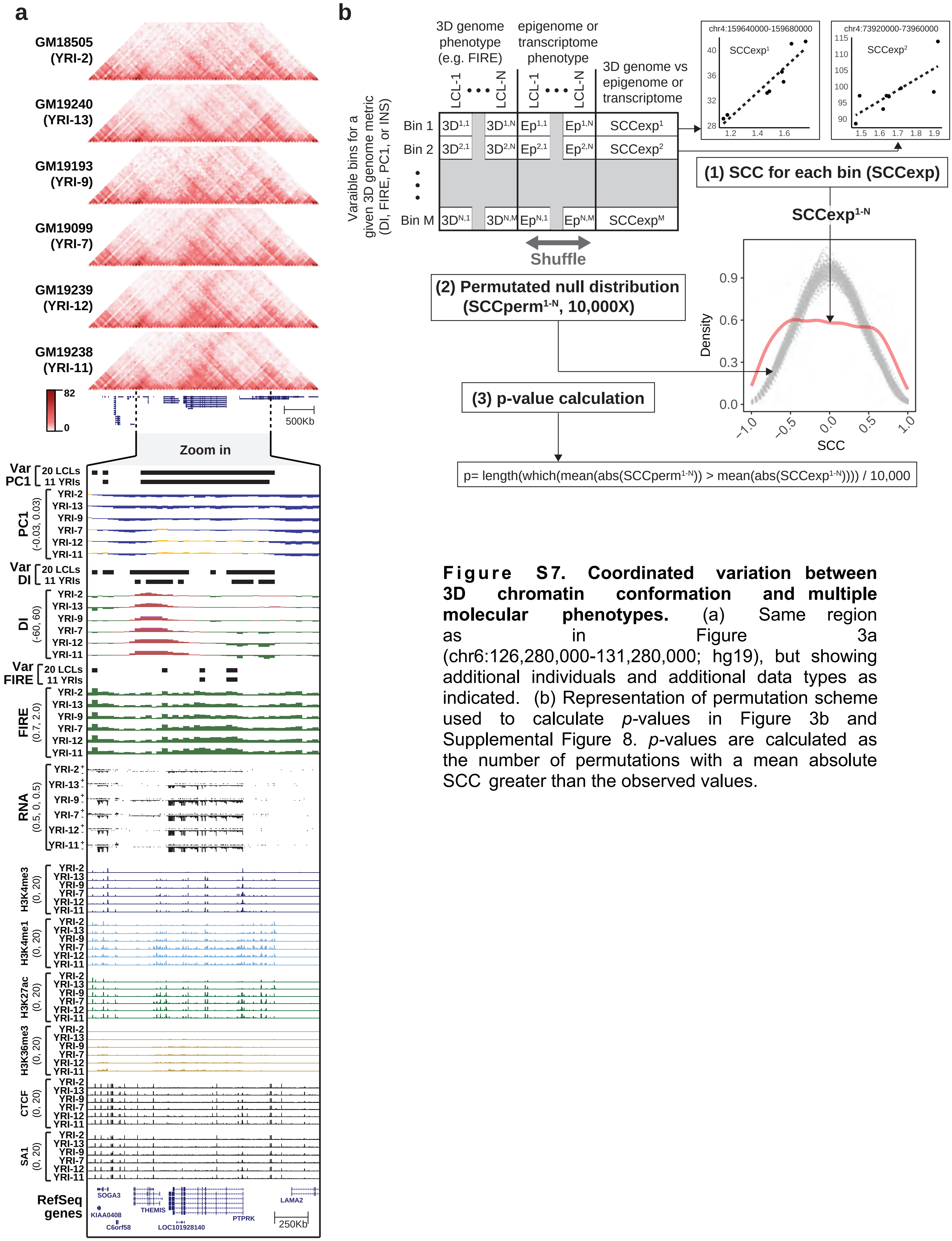

Figure S7. Coordinated variation between 3D chromatin conformation and multiple molecular phenotypes. (a) Same region as in Figure 3a (chr6:126,280,000-131,280,000; hg19), but showing additional individuals and additional data types as indicated. (b) Representation of permutation scheme used to calculate *p*-values in Figure 3b and Supplemental Figure 8. *p*-values are calculated as the number of permutations with a mean absolute SCC greater than the observed values.

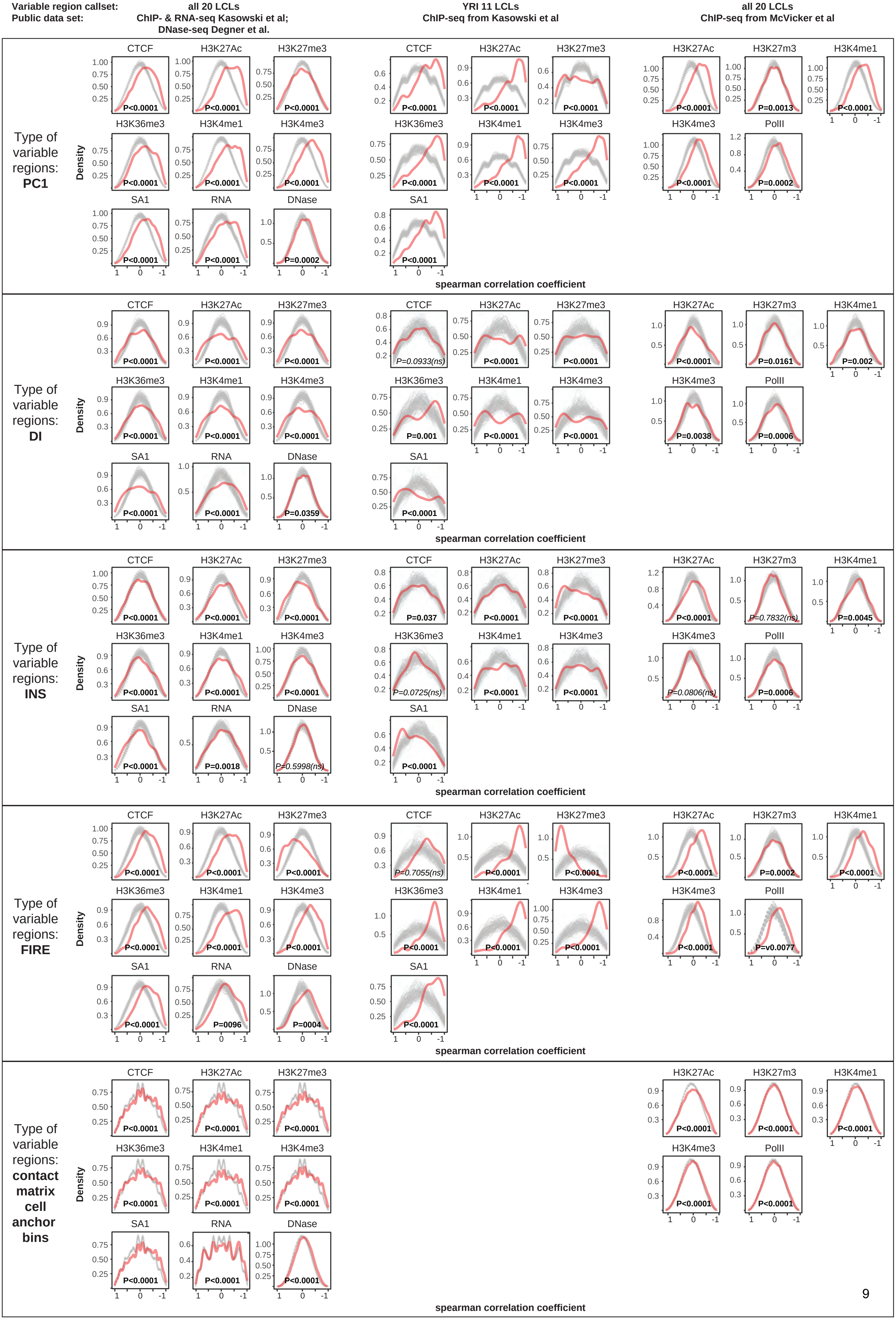
Variable region callset:
Public data set:
all 20 LCLs
ChIP- & RNA-seq Kasowski et al;
DNase-seq Degner et al.
YRI 11 LCLs
ChIP-seq from Kasowski et al
all 20 LCLs
ChIP-seq from McVicker et al
Type of variable regions: PC1
Type of variable regions: DI
Type of variable regions: INS
Type of variable regions: FIRE
Type of variable regions: contact matrix cell anchor bins
Density
CTCF
H3K27Ac
H3K27me3
H3K36me3
H3K4me1
H3K4me3
SA1
RNA
DNase
H3K27m3
PolII
spearman correlation coefficient
P<0.0001
P=0.0013
P=0.0002
P=0.0933(ns)
P=0.0161
P=0.002
P=0.001
P=0.0038
P=0.0006
P=0.0359
P=0.037
P=0.7832(ns)
P=0.0045
P=0.0725(ns)
P=0.0806(ns)
P=0.0018
P=0.5998(ns)
P=0.7055(ns)
P=v0.0077
P=0096
P=0004

Figure S8. Correlations between 3D genome phenotypes and other molecular phenotypes. Five subpanels show results for different types of variable regions: PC1, DI, INS, FIRE, contact matrix anchor bins (top to bottom). Density plots show the distribution of Spearman correlation coefficients (SCC) between 3D phenotype indicated to the left of each subpanel and molecular phenotypes as indicated above each plot. See Supplemental Figure 7b for schematic of calculations. Each subpanel is further organized into three columns as indicated at the top of the Figure: (left column) The ChIP-seq and RNA-seq data in this column are from Kasowski *et al*, 2013[2]. These data are the same as in Figure 3b, with the addition of histone modifications H3K4me1, H3K4me3, and H3K36me3, as well as DNase-seq data from Degner *et al*, 2012[7]. Plots include all individuals for which both Hi-C and the data type in question are available (N=8 individuals for ChIP-seq, N=7 for RNA-seq, and N=11 for DNase-seq). (middle column) SCC values with same ChIP-seq data as left column, but using a separate set of variable regions called in only 11 YRI individuals. We did not make these plots for variable contact matrix bins because there is only one variable bin call set. (right column) SCC using ChIP-seq data from McVicker *et al*, 2012[8]. These plots include N=10 individuals for which ChIP-seq and Hi-C data are available.

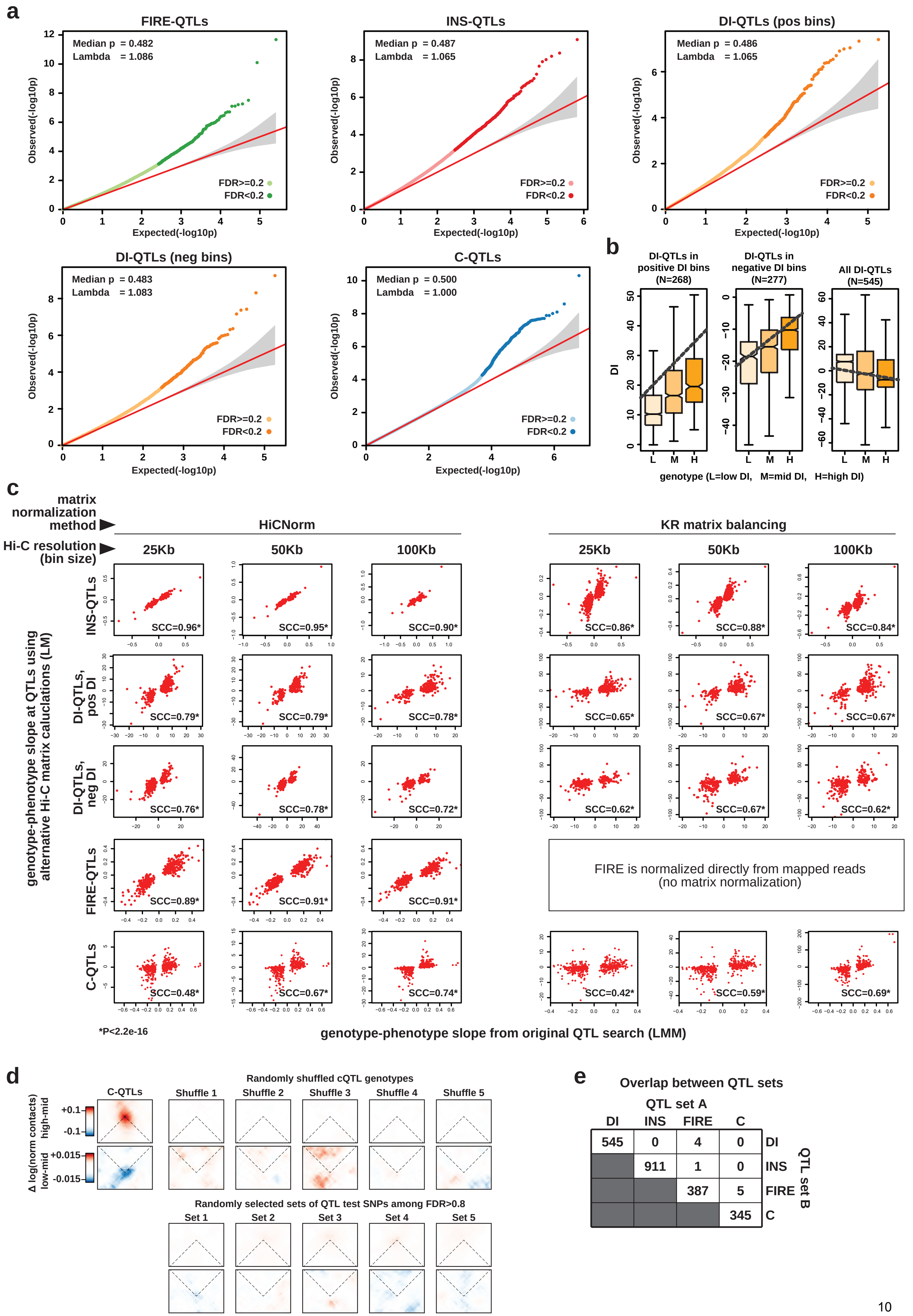
a
FIRE-QTLs
Median p = 0.482
Lambda = 1.086
FDR>=0.2
FDR<0.2
INS-QTLs
Median p = 0.487
Lambda = 1.065
DI-QTLs (pos bins)
Median p = 0.486
Lambda = 1.065
DI-QTLs (neg bins)
Median p = 0.483
Lambda = 1.083
C-QTLs
Median p = 0.500
Lambda = 1.000
Observed(-log10p)
Expected(-log10p)
b
DI-QTLs in positive DI bins (N=268)
DI-QTLs in negative DI bins (N=277)
All DI-QTLs (N=545)
DI
genotype (L=low DI, M=mid DI, H=high DI)
c
matrix normalization method
Hi-C resolution (bin size)
HiCNorm
KR matrix balancing
25Kb
50Kb
100Kb
INS-QTLs
DI-QTLs, pos DI
DI-QTLs, neg DI
FIRE-QTLs
C-QTLs
genotype-phenotype slope at QTLs using alternative Hi-C matrix caluclations (LM)
SCC=0.96*
SCC=0.95*
SCC=0.90*
SCC=0.86*
SCC=0.88*
SCC=0.84*
SCC=0.79*
SCC=0.79*
SCC=0.78*
SCC=0.65*
SCC=0.67*
SCC=0.67*
SCC=0.76*
SCC=0.78*
SCC=0.72*
SCC=0.62*
SCC=0.67*
SCC=0.62*
SCC=0.89*
SCC=0.91*
SCC=0.91*
FIRE is normalized directly from mapped reads (no matrix normalization)
SCC=0.48*
SCC=0.67*
SCC=0.74*
SCC=0.42*
SCC=0.59*
SCC=0.69*
*P<2.2e-16
genotype-phenotype slope from original QTL search (LMM)
d
Randomly shuffled cQTL genotypes
C-QTLs
Shuffle 1
Shuffle 2
Shuffle 3
Shuffle 4
Shuffle 5
Δ log(norm contacts)
high-mid
low-mid
+0.1
-0.1
+0.015
-0.015
Randomly selected sets of QTL test SNPs among FDR>0.8
Set 1
Set 2
Set 3
Set 4
Set 5
e
Overlap between QTL sets
QTL set A
QTL set B
DI INS FIRE C
DI 545 0 4 0
INS 911 1 0
FIRE 387 5
C 345

Figure S9. 3D chromatin QTLs. (a) QQ plots for each QTL search. In each QQ plot, the X-axis is the -log10 theoretical quantiles calculated from the uniform distribution. The Y-axis is the -log10 *p*-value calculated from linear mixed effects model for each type of QTL search. The grey area represents the 5% - 95% confidence bands based on Beta distribution Beta(i, M-i+1), where i is the i-th order statistics and M is the total number of tested SNPs. (b) DI values by genotype for bins with positive DI (left), negative DI (right), and all QTLs (right). A Simpson's paradox is observed when all bins are considered together. (c) Genotype-phenotype relationship at QTLs using Hi-C data binned at different resolutions (25Kb, 50Kb, 100Kb), and normalized with different methods (HiCNorm, Knight-Ruiz matrix balancing). X axes represent beta values from the original QTL search. Y axes represent beta value from a linear model fit to the genotype-phenotype relationship using Hi-C data of the indicated resolution and normalization method.(d) Aggregate contact plots as in Figure 4f, but including several negative control sets. Top row shows C-QTL SNPs with 5 random shuffles of the genotypes. Bottom row shows 5 random sets of non-QTL SNPs selected from the opposite end of the FDR spectrum from actual C-QTLs (FDR>0.8). (e) Number of direct overlaps between QTL sets.

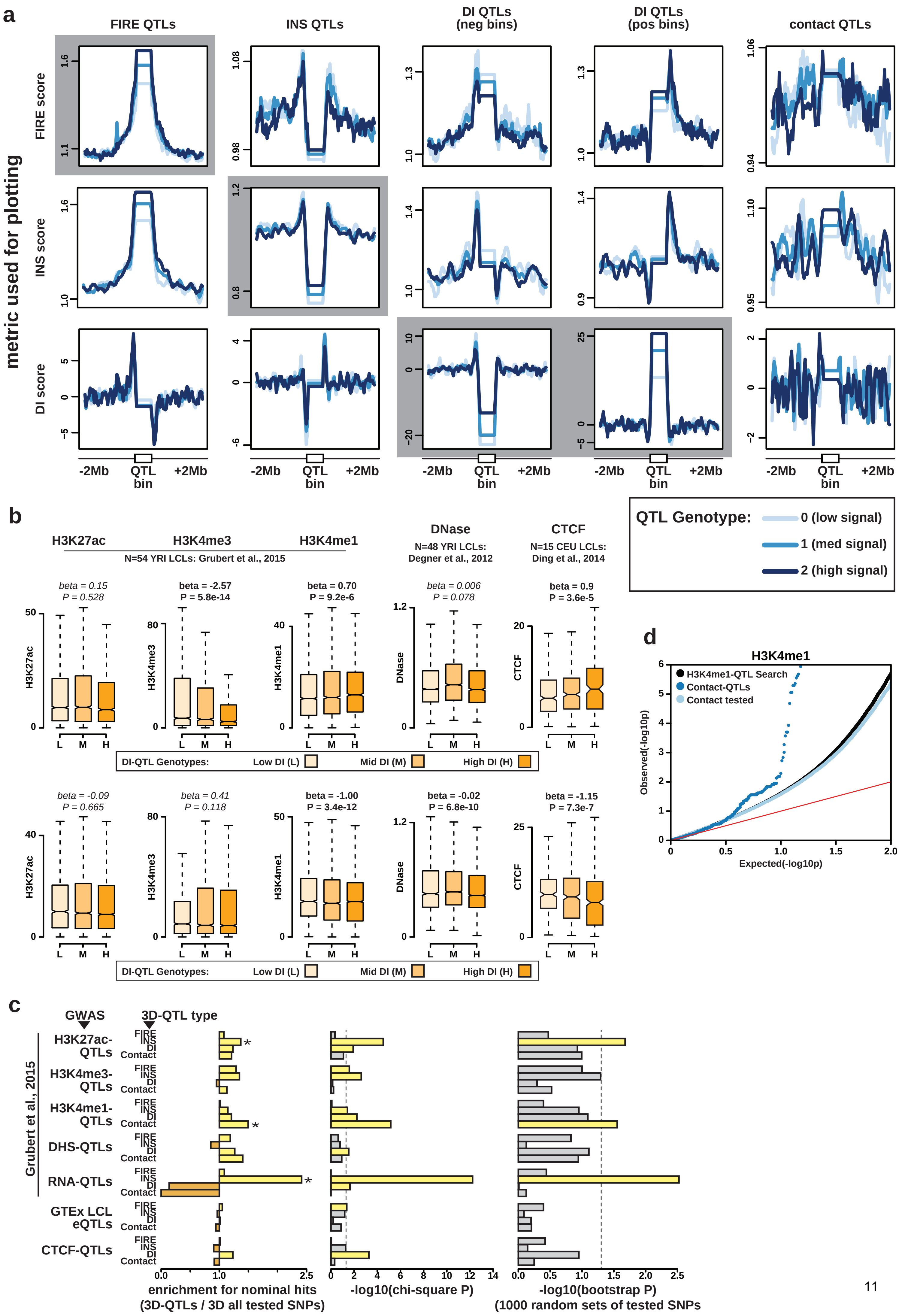
a
FIRE QTLs
INS QTLs
DI QTLs (neg bins)
DI QTLs (pos bins)
contact QTLs
metric used for plotting
FIRE score
INS score
DI score
-2Mb
QTL bin
+2Mb
QTL Genotype:
0 (low signal)
1 (med signal)
2 (high signal)
b
H3K27ac
H3K4me3
H3K4me1
N=54 YRI LCLs: Grubert et al., 2015
DNase
N=48 YRI LCLs: Degner et al., 2012
CTCF
N=15 CEU LCLs: Ding et al., 2014
beta = 0.15
P = 0.528
beta = -2.57
P = 5.8e-14
beta = 0.70
P = 9.2e-6
beta = 0.006
P = 0.078
beta = 0.9
P = 3.6e-5
DI-QTL Genotypes:
Low DI (L)
Mid DI (M)
High DI (H)
beta = -0.09
P = 0.665
beta = 0.41
P = 0.118
beta = -1.00
P = 3.4e-12
beta = -0.02
P = 6.8e-10
beta = -1.15
P = 7.3e-7
d
H3K4me1
H3K4me1-QTL Search
Contact-QTLs
Contact tested
Observed(-log10p)
Expected(-log10p)
c
GWAS
3D-QTL type
Grubert et al., 2015
H3K27ac-QTLs
H3K4me3-QTLs
H3K4me1-QTLs
DHS-QTLs
RNA-QTLs
GTEx LCL eQTLs
CTCF-QTLs
FIRE
INS
DI
Contact
enrichment for nominal hits
(3D-QTLs / 3D all tested SNPs)
-log10(chi-square P)
-log10(bootstrap P)
(1000 random sets of tested SNPs)

Figure S10. Influence of 3D chromatin QTLs on epigenomic and disease phenotypes. (a) Similar schema to Figure 4e, but showing FIRE, INS, DI score (indicated on the Y axis) as a function of genotype for each QTL set as indicated above each column. Grey boxes highlight the cases plotted in Figure 4e where the signal type and QTL set are the same. (b) Similar schema to Figure 5a, but showing DI-QTLs in positive bins (top) and negative DI bins (bottom). (c) Left subpanel shows the enrichment for 3D QTL SNPs with nominal significance in the indicated epigenetic or eQTL study calculated as follows: (fraction of indicated 3D QTL SNPs with nominal significance in the indicated molQTL study) / (fraction of SNPs tested in the indicated 3D QTL search with nominal significance in the indicated molQTL study). Asterisks mark values with $p<0.05$ by chi-square test (middle panel), and permutation test (right panel). Right panel shows the proportion of 1,000 random SNP subsets (selected from the tested SNPs) with enrichment values higher than the indicated true QTL set. Dotted lines mark $p<0.05$. (d) QQ plot shows the results of H3K4me1 QTL search from Grubert et al., with all tested SNPs shown as black points, and two subsets as follows: SNPs also tested in our C-QTL search (light blue), and SNP called as C-QTLs or in perfect LD with C-QTLs in the same 40Kb bin (dark blue).

1. Dixon, J. R. *et al.* Chromatin architecture reorganization during stem cell differentiation. *Nature* **518**, 331-336, doi:10.1038/nature14222 (2015).
2. Kasowski, M. *et al.* Extensive variation in chromatin states across humans. *Science* **342**, 750-752, doi:10.1126/science.1242510 (2013).
3. Hnisz, D. *et al.* Super-enhancers in the control of cell identity and disease. *Cell* **155**, 934-947, doi:10.1016/j.cell.2013.09.053 (2013).
4. McLean, C. Y. *et al.* GREAT improves functional interpretation of cis-regulatory regions. *Nat Biotechnol* **28**, 495-501, doi:10.1038/nbt.1630 (2010).
5. Ernst, J. *et al.* Mapping and analysis of chromatin state dynamics in nine human cell types. *Nature* **473**, 43-49, doi:10.1038/nature09906 (2011).
6. Ernst, J. & Kellis, M. ChromHMM: automating chromatin-state discovery and characterization. *Nature Methods* **9**, 215-216, doi:doi:10.1038/nmeth.1906 (2012).
7. Degner, J. F. *et al.* DNase I sensitivity QTLs are a major determinant of human expression variation. *Nature* **482**, 390-394, doi:10.1038/nature10808 (2012).
8. McVicker, G. *et al.* Identification of genetic variants that affect histone modifications in human cells. *Science* **342**, 747-749, doi:10.1126/science.1242429 (2013).
