# Supplementary material for: Common DNA sequence variation influences 3-dimensional conformation of the human genome
Source: Genome Biol. 2019 Nov 28;20:255. doi: 10.1186/s13059-019-1855-4 (PMC6883528; doi:10.1186/s13059-019-1855-4)
Supplement: Supplementary file 10 — Additional file 10. Review history. [file 13059_2019_1855_MOESM10_ESM.docx]

Review History

**First round of review**

**Reviewer 1**

**Are you able to assess all statistics in the manuscript, including the appropriateness of statistical tests used?**

yes

**Comments to author:**

  In this manuscript, Gorkin & Qiu et al. perform Hi-C on lymphoblastoid cell lines generated from 20 individuals from various backgrounds to identify the variability in 3D chromatin conformation.  They perform intensive computational analyses to intersect the Hi-C data with matched transcriptomic and other chromatin data such as ChIP-seq. Through several rigorous statistical analysis, the authors identify that several features of the higher-order chromatin structure, such as frequently interaction regions and insulation scores, differ between individuals and they are associated with QTLs, histone marks and transcriptional variability. They further show that these variable long-range interactions are highly enriched in disease associated regions.

    This study provides a nice and a timely example of an emerging theme of readily detectable inter-individual nuclear architecture, as assessed by Hi-C, and their interplay with other chromatin features such as QTLs, expression levels and histone marks. The data in this work appear to be sound, and exhaustive statistical analyses have been performed. However, there are certain parts of the manuscript in terms of scientific analyses which need to be revised. Apart from the major points about the analyses outlined below, the most important point is that the manuscript and the figures are very dense and hard to follow, especially starting from Figure 3, and thus requires some extensive re-wording, simplifying and spelling out several parts of the main text for a more general readership. Many parts in the main text pertaining the statistical analyses can actually be moved to the methods section for a smoother flow.

Major Comments:

1)     Throughout the manuscript, the authors analyze DI and INS values separately, where both values are used interchangeably by many publications to call TAD boundaries.  It is recommended that the authors call TAD boundaries by both methods, which should yield a highly overlapping TAD boundary list for each cell type, and present a single dataset throughout the figures labelled as "TAD Boundaries" along with PC1 and FIRE. Otherwise it is not clear what the differences between the DI and INS associations represent. 
2)     The authors have used an in dilution Hi-C protocol, which yields higher number of inter-chromosomal contacts than in-situ ligation. At large resolutions (>=250kb), can the authors identify related individuals from the same family or geography, by just clustering the variation in inter-chromosomal interactions? In other words, does the variability in 3D chromatin structure only exist in intra-chromosomal interactions, or is it also present in trans-chromosomal contacts as well? 
3)     According to the biological variability plot in Figure 3a, even though the fraction of matrix cells is higher in shorter genomic distances, it seems that there are more "intense" matrix cells as the genomic distance increases. It would be informative to plot the "intensity" of biological variability per distance, rather than the number of matrix cells depicted in Figure S6b, and 2f. 
4)     Related to the previous comment, the authors state that the matrix cells with biological variability tend to occur in groups or clusters. They should provide a plot with their sizes as well as their distances. Based on the scale presented in Figure 3a, or S5a, it is possible that these clusters can represent inter-compartment or inter-TAD interactions? 
5)     Although there is significantly more than expected overlap between the matrix cells and variable DI, INS, PC1 and FIRE regions, the overlap percentages shown in Figure 2g seem quite low (i.e <50%). The authors should show the correlation (e.g. in the form of a scatter plot) between the matrix cells versus the DI, INS, FIRE and PC1 variable regions. A 1D data type for the matrix cells or the variable regions can be generated by collapsing the matrix cell data, such as how frequent a region is involved in a biologically variable matrix cell within a 200kb window, similar to FIRE values. 
6)     In Figure 3b, S8 and S9, the authors show variable DI, INS, FIRE and PC1 value Spearman correlations with DNaseI, ChIP-seq and RNA-seq data. Although it is somewhat informative, the presentation of this data here is rather correlative. A more direct and a much informative way of presenting the data would be to show a scatter plot for each of DI, INS, FIRE and PC1 variable region values and the TPM values of RNA-seq or the normalized read counts of DNaseI or ChIP-seq data. Plotting the same scatterplots for non-variable regions (i.e rest of the genome or size-matched regions) could be used as a control. Although partially touched upon in Figure 5, this way, the reader could visually obtain two pieces of information that could not be interpreted with the initial analysis: i) the extent of change in the variable regions. A change in INS would indicate the strength of a TAD boundary and a change in PC1 would indicate the compartmentalization status, which biologically would indicate different mechanisms. ii) their association with the level of values of RNA-seq and chromatin data. 
7)     In Figure 4f, the authors show the normalized contacts for c-QTLs. A set of same number of QTLs at non-variable regions should be included as a control (background). 

Minor Comments:
1)     On line 255, the authors state "Moreover, the correlations between multiple molecular phenotypes at the same region suggest that shared mechanism(s) underlie variation in these phenotypes across individuals.". It is not clear what "shared mechanisms" are depicted. Can the authors also comment on whether it is the variation in higher-order structure that leads to variation in expression, chromatin marks or even QTLs or vice versa? 
2)     It would be interesting to check, if there is sufficient analysis power, whether there are any transcription factor (TF) motifs other than CTCF enriched in variable regions (either matrix cells, DI, INS, FIRE or PC1), and whether there is an evidence of a QTL disrupting or creating a binding motif and accordingly a long-range interaction is emerged or perturbed, independent of CTCF. 
3)     On line 304, "Simpson's paradox" and "genotype trend" should be defined.
4)     Supplementary Figures S5E and S6C are hard to interpret and follow.
5)     A final model figure depicting the key findings of this study would be beneficial.

**Reviewer 2**

**Are you able to assess all statistics in the manuscript, including the appropriateness of statistical tests used?**

yes

**Comments to author:**

The paper studies variation in HiC maps across individuals, and collects unique data for that purpose. It observed inter-individual differences, and highlights their association to molecular observation and overlap with disease trait loci. The dataset collected as part of this work is impressive. Yet the primary claim of genetic association is not supported, requiring massive re-analysis and re-writing.

Major comments:
1. The analysis of replicates vs. inter-individual variability (Fig 1-2-3) only proves individual contribution to variance, not genetic variance. Heritability of the traits analyzed would go a long way to support the genetic claims, but as the data stand right now, they are consistent with issues with the processing of each individual's material that cause variability. 

2. The association analysis is flawed. The QQ-plots invalidate all analyses besides C-QTLs as overinflated. Furthermore, the replication data does not actually replicate the results. Of the 300-1100 hits in the discovery data, if indeed FDR<.2, then >80% of them are real. These show LOD of 3-12. With such strong signals in the discovery set, the power for these hits to achieve the right direction of effect, i.e. LOD of 0.3, is overwhelming, and the results should be much stronger. 

Minor comments:

3. Page 11: need to be reanalyzed using a completely non overlapping set of individuals
4. PC1 is a terrible phenotype, because it flips at TAD boundaries. When a TAD boundary is removed for an individual, e.g by removal of CTCF binding, PC1 Will be flipped from that point onwards. For cis analysis, this is a nightmare.
5. Line 832-838 The filtering step means that PC1 Is not a pure result of the HiC experiments, but rather integrate gene density. Since gene-density of chromosomes is not independent of functional activity, this renders the results in Fig 3 questionable.
6. Line 838-839 Manual inspection as a QC step is unacceptably irreproducible. If there are QC analysis or inspection steps, they should be coded up as computer instructions. These can be developed from manually obtained insight on the discovery samples, but not on samples considered as replication/validation.
7. Fig 1a 2a 3a: In the red LD triangle plots: color scale should be the same for all samples' 
8. Fig 1c: the right panel should be removed. Everybody is reading this electronically, and can zoom in 3x if they want to.
9. Fig 1d is misleading as the significance threshold is not Bonferroni-corrected for the multiple testing of N=50 bars. The corrected threshold should be added, at -log10(p)=3. I agree with the authors that even at the relaxed threshold of nominal significance, -log10{p) = 1.3, as shown, there is enrichment of significant bars at each of the four 2.5Mb windows shown, but the bar-chart is still misleading to think each of the yellow bars is significant.
10. Fig1b: The Wilcoxon score shortchanges the significance. For INS and DI, the box-and-whiskers suggest that the top quartiles of the 20 within coefficients are higher than all of the 760 between coefficients. The p-value just for that is 20*19*18*17*16/(780*779*778*777*776) < 10^-13 . 
11. Limma is an expression data analysis package. It is not clear why this is the tool of choice to analyze 3D genome structure data. While statistics are statistics, no matter what, Limma incorporates normalizations, assumptions and distributions that are specific to RNAseq data, so one would need reassurances these are not unduly changing the results.
12. Fig 2: the presentation of a new set of 4 individuals is cherrypicking, which is an attempt to mislead. If the claims are correct, the authors should be able to demonstrate them with any four samples. They should stick to YRI-1,2,3 and 4.
13. Power analysis for detecting effects at p=0.05 is irrelevant as this is not the analysis that actually takes place
14. L799 ration -> ratio

**Reviewer 3**

**Are you able to assess all statistics in the manuscript, including the appropriateness of statistical tests used?**

yes

**Comments to author:**

This is a very nice study, carefully done and expertly analyzed. I reviewed the statistical analysis carefully, and was pleased to note the attention to details, including batch and confounder correction, distinction between correlation and cause, and proper hypothesis driven tests. My comments are truly minor, with the exception of the first one:

Because of the small sample size, I am troubled by the confounding effects of population and potential other correlates on the QTL analysis. I suggest to remove the data from the samples that are not Yoruban individuals and confirm that the QTL analysis is robust. One way to test for this is to check that pairs of individuals do not share QTL genotypes more often than expected by chance (namely, that there is no unexpected structure in the QTL data).

Much more minor comments:

It is not clear to me how they used limma to perform F tests (comparisons of variation within and between individuals).

I wonder how robust are the QTLs you identified to other approaches to characterize the Hi-C data (for example, if you analyzed the contact frequencies by using another normalization method). 

Similarly, I wonder about the robustness of the results with respect to different data resolution. 

It was not clear to me how replicates were considered in the QTL analysis.

**Authors Response**

**Point-by-point responses to the reviewers’ comments:**

*We thank the reviewers for their constructive feedback. We made major changes in response to their comments. We hope the referees will agree that their input has contributed to significant improvements over the previous version. Please find our point-by-point responses in bold below.*

**Reviewer #1:**

In this manuscript, Gorkin & Qiu et al. perform Hi-C on lymphoblastoid cell lines generated from 20 individuals from various backgrounds to identify the variability in 3D chromatin conformation. They perform intensive computational analyses to intersect the Hi-C data with matched transcriptomic and other chromatin data such as ChIP-seq. Through several rigorous statistical analysis, the authors identify that several features of the higher-order chromatin structure, such as frequently interaction regions and insulation scores, differ between individuals and they are associated with QTLs, histone marks and transcriptional variability. They further show that these variable long-range interactions are highly enriched in disease associated regions.

This study provides a nice and a timely example of an emerging theme of readily detectable inter-individual nuclear architecture, as assessed by Hi-C, and their interplay with other chromatin features such as QTLs, expression levels and histone marks. The data in this work appear to be sound, and exhaustive statistical analyses have been performed. However, there are certain parts of the manuscript in terms of scientific analyses which need to be revised. Apart from the major points about the analyses outlined below, the most important point is that the manuscript and the figures are very dense and hard to follow, especially starting from Figure 3, and thus requires some extensive re-wording, simplifying and spelling out several parts of the main text for a more general readership. Many parts in the main text pertaining the statistical analyses can actually be moved to the methods section for a smoother flow.

*We thank the reviewer for commenting on the rigor and timeliness of the work, as well as the shortcomings of the manuscript in its previous form. We have extensively revised the text and figures for improved content and a smoother flow, particularly in the sections pointed out by the reviewer.*

Major Comments:

1) Throughout the manuscript, the authors analyze DI and INS values separately, where both values are used interchangeably by many publications to call TAD boundaries. It is recommended that the authors call TAD boundaries by both methods, which should yield a highly overlapping TAD boundary list for each cell type, and present a single dataset throughout the figures labelled as "TAD Boundaries" along with PC1 and FIRE. Otherwise it is not clear what the differences between the DI and INS associations represent.

*We agree with the reviewer that as originally written it is not clear why we analyzed DI and INS separately, rather than just using TAD boundaries. We now include TAD boundary calls for each LCL as Supplemental Table 4. To further address the reviewer’s concern, we have elaborated in the text (lines 124-131) as well as below about our reasons for including both DI and INS as quantitative phenotypes, which we believe adds value to the manuscript beyond presenting only TAD boundaries. Our rationale is as follows: • DI and INS are quantitative phenotypes that can be measured across the entire genome, whereas the presence of a TAD boundary is a binary phenotype (much like a peak call from ChIP-seq data) that does lend itself easily to quantitative comparisons of variability.*

*TAD boundary calling (much like peak calling) is highly dependent on the algorithm and specific parameters used (Ref: https://genomebiology.biomedcentral.com/articles/10.1186/s13059-018-1596- 9), and in our experience there is little consensus in the field currently about which approach is best.*

*• We agree with the reviewer that DI and INS are related phenotypes, but they are not the same. INS directly measures contact insulation (the defining characteristic of TAD boundaries), whereas DI measures imbalance of contact directionality which can occur adjacent to TAD boundaries but can also occur elsewhere. TAD boundaries show an abrupt shift from strong upstream DI to strong downstream DI. However, strong directional bias as measured by DI can also occur within TADs. For example, we see anecdotally that promoters often show either upstream or downstream DI, likely reflecting regulatory element interactions and/or transcription.*

*• We find that the variable regions and QTLs for these two phenotypes, although overlapping in some cases, are largely distinct (Figures S5d, S9e)*

*For these reasons, we believe that it is informative to include both phenotypes in the manuscript. We hope the additional data and explanation make the similarities and differences more apparent.*

2) The authors have used an in dilution Hi-C protocol, which yields higher number of inter-chromosomal contacts than in-situ ligation. At large resolutions (>=250kb), can the authors identify related individuals from the same family or geography, by just clustering the variation in inter-chromosomal interactions? In other words, does the variability in 3D chromatin structure only exist in intra-chromosomal interactions, or is it also present in trans-chromosomal contacts as well?

*The reviewer raises an interesting point. Using principal component analysis with contacts binned at very large resolution (1Mb bins) we do see some clustering by ethnicity in trans contacts, similar to what we see with cis contacts (please see plots below). Throughout the manuscript we avoided analyses based on trans contact and focus on cis contacts for two main reasons: (1) Technical – at least some of the additional trans interactions seen in “dilution” vs “in situ” Hi-C likely reflect technical noise arising from spurious inter-molecular ligation products in solution. (2) Biological – the positioning of chromosomes relative to each other is highly variable among individual cells, even in an ostensibly homogeneous cell population. We don’t doubt that there are tendencies for certain chromosomes to end up near each other, as well as potentially functional trans interactions. However, they are likely to be far less frequent that cis interactions in a population of cells and thus hard to detect and reproduce. For these reasons we are not sure how to interpret observations made from trans contacts, and have not included them in the manuscript.*

3) According to the biological variability plot in Figure 3a, even though the fraction of matrix cells is higher in shorter genomic distances, it seems that there are more "intense" matrix cells as the genomic distance increases. It would be informative to plot the "intensity" of biological variability per distance, rather than the number of matrix cells depicted in Figure S6b, and 2f.

*We agree that this is informative. We have added the plots described (biological variably vs distance) to Figure S6e. We find that the average biological variability scales with distance in a similar manner to the fraction of variable cells in Figure 2f and S6d, but we note there is high variability.*

4) Related to the previous comment, the authors state that the matrix cells with biological variability tend to occur in groups or clusters. They should provide a plot with their sizes as well as their distances. Based on the scale presented in Figure 3a, or S5a, it is possible that these clusters can represent inter-compartment or interTAD interactions?

*We agree that more detail about the clusters of biologically variable matrix cells would be useful. We now include an analysis in Figure S6g that quantifies, as a function of distance, the average number of bins neighboring a variable bin that also themselves variable. Regarding inter-compartment and interTAD interactions, we do not find evidence that variable bins tend to represent these types of interactions. These results are now included in Figure S6h-i.*

5) Although there is significantly more than expected overlap between the matrix cells and variable DI, INS, PC1 and FIRE regions, the overlap percentages shown in Figure 2g seem quite low (i.e <50%). The authors should show the correlation (e.g. in the form of a scatter plot) between the matrix cells versus the DI, INS, FIRE and PC1 variable regions. A 1D data type for the matrix cells or the variable regions can be generated by collapsing the matrix cell data, such as how frequent a region is involved in a biologically variable matrix cell within a 200kb window, similar to FIRE values.

*This is a good point. The way we presented the overlap percentages was not ideal. We showed the raw overlap percentage – i.e. the percentage of all variable matrix cell anchor bins that were also called as variable bins for DI/INS/FIRE/PC1. However, not all variable matrix cell anchor bins were tested for variable DI/INS/FIRE/PC1. When we re-run these analyses including only variable matrix cell anchor bins that were also tested for variable DI/INS/FIRE/PC1, the overlap percentages are much higher, and have been added to Figure 2g. The overlap percentages are still <50%, but this seems to be an arbitrary cutoff, and we are not sure that a higher expectation is warranted. For example, a single variable matrix bin or even a small cluster of variable bins may not change dramatically enough or in the right pattern to also yield variable DI/INS/FIRE/PC1 bins. On the other hand, when we see variable DI/INS/FIRE/PC1 bins we would expect that in most cases there would be one or more variable matrix cell(s) with those anchor bins. Indeed, the overlap percentages of variable DI/INS/FIRE/PC1 bins with variable matrix cell anchor bins are all well over 50%, and have been added to Figure S6f.*

*We also appreciate reviewer’s suggestion of a scatterplot, but there are two obstacles that prevent this: (1) Although we can “collapse” the matrix cell data to a 1D list of anchor bins, we cannot assign each anchor bin a 1D value that accurately captures the contact frequency of the matrix cells involving a given anchor bin. Simple summary statistics such a mean, median, etc do not work well because contact frequency is highly dependent on contact distance and this dimension is lost when collapsing contact frequency values to a single summary statistic. DI/INS/FIRE/PC1 are themselves 1D metrics used to capture various aspects of the contact matrix by collapsing values in different ways. However, we think*

*that deriving a new 1D phenotype metric is beyond the scope of our study, and would not achieve the goal of comparison between variable bins and variable matrix cell anchor bins. (2) To make a correlation scatterplot between two different 1D metrics (e.g. FIRE and DI) for a set of variable bins we would need one data point per bin. However, in our data, we have 20 data points per bin (one data point per individual per bin). We can make* *a scatterplot for a single region that shows the correlation across individuals, but the range of the axes are different for each region, and thus if we try to plot all points for all regions on the same axes the patterns are obscured. We thought a lot about this plotting problem both in the original submission and in revision. We agree with the reviewer that, where possible, scatterplots are an intuitive way to visualize correlative data. However, in our case the higher dimensionality of the data prevents summary in a straightforward scatterplot. We elaborate on this point with examples in response to point #6 below.*

6) In Figure 3b, S8 and S9, the authors show variable DI, INS, FIRE and PC1 value Spearman correlations with DNaseI, ChIP-seq and RNA-seq data. Although it is somewhat informative, the presentation of this data here is rather correlative. A more direct and a much informative way of presenting the data would be to show a scatter plot for each of DI, INS, FIRE and PC1 variable region values and the TPM values of RNA-seq or the normalized read counts of DNaseI or ChIP-seq data. Plotting the same scatterplots for non-variable regions (i.e rest of the genome or size-matched regions) could be used as a control. Although partially touched upon in Figure 5, this way, the reader could visually obtain two pieces of information that could not be interpreted with the initial analysis: i) the extent of change in the variable regions. A change in INS would indicate the strength of a TAD boundary and a change in PC1 would indicate the compartmentalization status, which biologically would indicate different mechanisms. ii) their association with the level of values of RNA-seq and chromatin data.

*We appreciate the reviewer’s point and suggestions about more informative ways to plot this type of data. As touched on in our response to point 5 above, the dimensionality of our data prevents summary in a straightforward scatterplot. In such a scatterplot we would ideally have one dot per region. However, our data has one dot per individual per region (i.e. 20 dots per region), and when all regions are plotted on the same scatterplot the additional information suggested by the reviewer (i.e. extent of change at each region) is obscured. For example, below we show 5 regions with strong correlation between FIRE and H3K27ac across individuals. Note that the axes values vary dramatically between plots (particularly the y-axis). Thus, when we try to plot many regions in a single scatterplot we get a cloud that is hard to interpret. Due to Simpson paradox, the correlation calculated from the combined data may be different from the correlation calculated from each region. This problem is further exacerbated when we consider a phenotype like DI where there is no a priori expectation of the direction of correlation between DI and CTCF, for example. In this case, we would see an abundance of both positive and negative slopes, which would be hard to see clearly in an aggregate scatter or line plot. In our opinion, the SCC density plots in Figure 3b and S8 are a better way to visualize this relationship because you can easily see shoulders on both ends of the SCC distribution. We have heavily revised the schematic in Figure S7b to better illustrate this point, and guide readers through these figures.*

*The reviewer’s point is well taken that the SCC density do not convey the magnitude (i.e. slope) of the correlations, which would be valuable to know. However, we think our data is not ideal for quantifying the strength of the relationship between 3D phenotypes and epigenomic/transcriptomic phenotypes across individuals, because of the limited resolution of the Hi-C data and small number of individuals. Our conclusion from these data is simply that there are relationships between 3D phenotypes and epigenomic/transcriptomic phenotypes across individuals, and we think the evidence presented here to support that conclusion is quite robust. We do not use these data to draw conclusions about the*

*magnitude of these relationships; only the magnitude of statistical confidence with which we can say they exist.*

7) In Figure 4f, the authors show the normalized contacts for c-QTLs. A set of same number of QTLs at non-variable regions should be included as a control (background).

*We see the reviewer’s point that it would be helpful to have controls for the plot in Figure 4f. We do not think that “QTLs at non-variable regions” would be the best negative controls because (a) our QTL search was targeted to variable regions and thus we do not have QTL calls in non-variable regions; (b) significant QTLs even in non-variable regions (if we had to searched for them) would be expected to show some genotype-dependent contact frequency so they might not serve well as negative controls.*

*We now provide two types of negative controls for the 4f plot in Supplemental Figure 9d: (1) our actual C-QTLs but with the genotypes randomly shuffled to disrupt the link between genotype and contact frequency (we provide plots for 5 random genotype shuffles); (2) size-matched random sets of non-QTL SNPs (i.e. SNPs tested in the QTL search but selected from the opposite end of the FDR spectrum). We now provide 5 random sets of SNPs selected from the QTL test set with FDR >0.8.*

Minor Comments:

1) On line 255, the authors state "Moreover, the correlations between multiple molecular phenotypes at the same region suggest that shared mechanism(s) underlie variation in these phenotypes across individuals.". It is not clear what "shared mechanisms" are depicted. Can the authors also comment on whether it is the variation in higher-order structure that leads to variation in expression, chromatin marks or even QTLs or vice versa?

*We agree that this is an important question, and have added additional discussion of this point to the text. Unfortunately, we don’t think our data can distinguish between these two possibilities. In the original text we had narrative on this topic in the results section, but in this revision we have moved it to the discussion section where it is probably a better fit. That section now reads:*

*“Another key finding of our study is that regions which vary in 3D chromatin conformation across individuals also tend to vary in measures of transcriptional and regulatory activity. This supports the existence of shared mechanisms that underlie variation in 3D chromatin conformation, transcription, and epigenomic properties. Our data does not resolve whether variation in higher-order chromatin conformation leads to variation in gene expression and histone modification state, or vice versa. However, we suspect that no single mechanism or causal hierarchy applies to all regions of the genome with variation in one or more of these properties.” (lines 421-428)*

2) It would be interesting to check, if there is sufficient analysis power, whether there are any transcription factor (TF) motifs other than CTCF enriched in variable regions (either matrix cells, DI, INS, FIRE or PC1), and whether there is an evidence of a QTL disrupting or creating a binding motif and accordingly a long-range interaction is emerged or perturbed, independent of CTCF.

*We agree with the reviewer that it could be very interesting to examine motifs beyond CTCF. We were not able to do this analysis in our study for a few reasons: (1) The variable regions are 40Kb each, and thus it would not be feasible to perform a motif enrichment analysis in these regions. Even if we further limited to ChIP-seq peaks within variable regions, we expect that many peaks in the analysis would be unrelated to the variability and introduce noise into the analysis because of limited resolution of the Hi-C data. We think results of such an analysis would be difficult to interpret. (2) In the case of the QTLs, LD prevents us from identifying the functional variant(s) underlying each association that could be disrupting TFs. Hi-C resolution is also a problem here, because we selected at most one QTL SNP per 40Kb bin, often among several in perfect LD. Even in well-powered GWAS not limited by Hi-C (or other) data resolution, we would probably need additional fine-mapping to get to the level of individual functional variants and potentially disrupted TF motifs. (3) For the CTCF motif analysis, we used a curated set of occupied convergent CTCF motifs in loop anchors identified in much higher resolution Hi-C data form GM12878 (Rao et al., 2014). This was a unique dataset that enabled the targeted analysis of loop anchor CTCF motifs in Figure 4.*

*In response the reviewer’s comment, we attempted to perform similar analysis as in Figure 4 but for additional motifs. For these additional motifs we chose PU.1, IRF factors, and NF-κb because of their enrichment in LCL open chromatin (not shown), and the known roles of those factors in lymphocyte biology. To reproduce the Rao et al approach to identifying occupied loop anchor CTCF motifs we identified instances of these other motifs in Rao et al loop anchors that also overlapping ChIP-seq peak for one or more of the corresponding TFs (please see Figure panel below). Once identifying occupied loop anchor motif instances, we then performed the same analysis as in Figure 4 looking at the effect of “motif-disrupting” SNPs on loop strength. The results are shown below, but are inconclusive. The CTCF analysis shows significant linear association between genotype and loop strength, which is highly localized to the loop bin. Part of the problem is that, relative to the CTCF analysis, these other analyses are severely underpowered because the number of anchor motifs and SNP therein are much lower than for CTCF. We do not observe this type of clear genotype-dependent effect on loop strengths for any of*

*these other factors. The note that the trend for IRF factors is statistically significant considering only the loop bin (right-most column above), but the heatmap shows that the loop bin does not stand out in this region, and suggests that this likely noise due the very small sample size of 15 motif disrupting SNPs. We did not include these results in the manuscript because we think the lack of power makes them difficult to interpret.*

3) On line 304, "Simpson's paradox" and "genotype trend" should be defined.

*We have added definitions in lines 305-306.*

4) Supplementary Figures S5E and S6C are hard to interpret and follow.

*We see that reviewer’s point that these mosaic plots are not intuitive and have removed them.*

5) A final model figure depicting the key findings of this study would be beneficial.

*We agree with the reviewer’s point. We now include a model/summary as Figure 6.*

**Reviewer #2:**

The paper studies variation in HiC maps across individuals, and collects unique data for that purpose. It observed inter-individual differences, and highlights their association to molecular observation and overlap with disease trait loci. The dataset collected as part of this work is impressive. Yet the primary claim of genetic association is not supported, requiring massive re-analysis and re-writing.

*We thank the reviewer for recognizing the value of the dataset and for constructive feedback about how to improve our results, which we have taken many steps to incorporate as described point-by-point below. However, we respectfully disagree with the reviewer’s assertion that our conclusion of genetic influence of 3D chromatin architecture is not supported. We would like to point out (1) the effect of CTCF motif disrupting SNPs on Hi-C contacts in Figure 4a-d. We note that this analysis is independent of any QTL search; (2) The QTLs we identify show consistent direction of effect in aggregate in an independent set of six individuals (5/6 from a different population than the discovery set individuals). 3) We find consistent genotype-phenotype relationships at the QTLs in orthogonal epigenomic datasets generated by other labs on material isolated independently, from different LCL/individuals.*

Major comments:

1. The analysis of replicates vs. inter-individual variability (Fig 1-2-3) only proves individual contribution to variance, not genetic variance. Heritability of the traits analyzed would go a long way to support the genetic claims, but as the data stand right now, they are consistent with issues with the processing of each individual's material that cause variability.

*We agree with the reviewer and do not claim that the inter-individual variation described in Figures 1-3 is necessarily genetic. Indeed we suspect that much of this variation is not due to genetics. We now state this more clearly in the results section (lines 151-153), and have added a more detailed discussion of this point to the discussion section (lines 421-428). These analyses were intended to characterize the variability itself, before assessing the role of genetics. We expect that these variable 3D genome regions and their relationship to other epigenomic and transcriptomic features are of interest to chromatin biologists even in the absence of genetic influence.*

*We also acknowledge that technical artifacts related to sample processing can cause variability, as the reviewer states. However, our data shows that this is probably not a major factor in the variability we observed because the variable 3D chromatin regions we identified are correlated with variability in multiple epigenomic and transcriptomic features assayed by multiple other groups on samples grown and processed independently (Figure 3b).*

2. The association analysis is flawed. The QQ-plots invalidate all analyses besides C-QTLs as overinflated. Furthermore, the replication data does not actually replicate the results. Of the 300-1100 hits in the discovery data, if indeed FDR<.2, then >80% of them are real. These show LOD of 3-12. With such strong signals in the discovery set, the power for these hits to achieve the right direction of effect, i.e. LOD of 0.3, is overwhelming, and the results should be much stronger.

*We see the reviewer’s point that there is some global inflation apparent in the QQ plots. However, we respectfully disagree that this invalidates our results. The lamba gc values (i.e. genomic inflation factors) for our QTL searches are in line with or better than many published and highly-regarded molecular QTL studies (see plot below). Our lambda values are all under 1.1, which in our experience is often regarded as a reasonable threshold. Moreover, some level of inflation is expected here (i.e. higher than in a classic GWAS) because we performed a targeted search within variable regions that are likely to be enriched for real QTL SNPs relative to the whole-genome background. In addition, our individual tests are not truly independent because 3D chromatin phenotypes can involve large regions, and there is extensive LD in our small sample size (11 YRI individuals used in the QTL search). We have tried to mitigate these factors to some extent in our final QTL counts by allowing at most one QTL for each 40Kb bin in a given search.*

*We have performed several additional analyses summarized below to rule out major confounding factors that would need to be further accounted for in the QTL search:*

*• We added the first three PCs calculated from the genotype matrix as covariates in our LMM model to control for potential cryptic relatedness or population substructure (top row of figure below).*

*• We added the first three PCs calculated from the phenotype matrix as covariates in our LMM model to control for potential batch effects (middle row of figure below).*

*• We also re-normalized each 1D phenotype (i.e. DI, FIRE, INS) using ComBat (PMID: 16632515) to regress out the impact of Hi-C processing batches (bottom row of figure below).*

*In all cases, we did not observe reduction in the QQ-plot inflation levels. This suggests that the observed inflation is not likely caused primarily by population substructure or batch effects. Given that the inflation observed in the original QTL search is not significantly improved with these methods, is within a reasonable range, and yields QTLs that are supported by data from additional individuals and orthogonal epigenetic and transcriptomic datasets, we have not replaced the original QTL results.*

*We agree with the reviewer that the list of QTLs reported here is not comprehensive and contains false positives. However, we think our data clearly shows that these QTL sets contain many true positives. We revised the discussion accordingly (lines 411-420). We would like to emphasize here that our main conclusion from these data is not the QTL themselves, but rather, the demonstration that common sequence variation does influence 3D chromatin conformation. This has not been shown previously, and is important because it points to 3D chromatin conformation as a potential molecular mechanism that can mediate disease risk. We expect that future studies with more individuals and higher-resolution data will produce much more comprehensive QTL lists. We think that our study is a critical step toward those efforts by providing rationale and demonstrating feasibility.*

*With regard to replication, we emphasize that we are attempting to replicate between populations, and that our validation individuals are a mixture of 4 different populations (Table S2; YRI N=1, CEU N=1, CHS N=2, PUR N=2). This obviously impacts the replication ability, in addition to the small sample size and noise in the Hi-C data.*

Minor comments:

3. Page 11: need to be reanalyzed using a completely non overlapping set of individuals

*We removed overlapping individuals and re-made Figures 5a-b and S10b for the H3K27ac, H3K4me3, H3K4me1, and DNase analysis. There are no overlapping individuals for CTCF, so those data are unchanged. Text and figures have been updated accordingly.*

4. PC1 is a terrible phenotype, because it flips at TAD boundaries. When a TAD boundary is removed for an individual, e.g by removal of CTCF binding, PC1 Will be flipped from that point onwards. For cis analysis, this is a nightmare.

*PC1 (i.e. derived from PCA of Hi-C contact maps) is a well-established phenotype in 3D chromatin architecture reflecting the so-called “A” and “B” compartments of chromatin in the nucleus (PMIDs: 19815776, 23473598, 27437574, 28525758). We respectfully disagree that PC1 is a nightmare for cis analysis. On the contrary, such analysis is standard in the field. Moreover, our analysis in Figure 3b shows that variability in PC1 in correlated with independently measured changes in epigenomic and transcriptomic properties, further supporting the validity of the variable PC1 regions we have identified.*

*Regarding the concern about flipping, we are not sure what the reviewer is referring to here. We are not aware of any scenario in which PC1 flips as a result of a TAD boundary deletion and remains “flipped from that point onwards.” We and others have shown that a given TAD or other region can change compartments from A to B or vice-versa during differentiation (PMID: 25693564), which is reflected in a “flip” in the sign of PC1 (i.e. negative or positive) at that region only. However, those flips do not propagate throughout the rest of the chromosome. They reflect the switch of a region of chromatin from one compartment to another.*

*We would also like to point out that PC1 is not part of our QTL analysis, and thus has no bearing on our findings of genetic influence on 3D chromatin organization.*

5. Line 832-838 The filtering step means that PC1 Is not a pure result of the HiC experiments, but rather integrate gene density. Since gene-density of chromosomes is not independent of functional activity, this renders the results in Fig 3 questionable.

*The lines referenced by the reviewer don’t match up with the PC1 methods section in our copy, but we assume that the reviewer is referring to the use of gene density with respect to PC1 (i.e. compartmentalization) phenotyping. We have clarified our use of gene density in the compartment phenotyping in the methods section. We did not use gene density in the actual PCA calculations. Rather, we used gene density to orient the otherwise arbitrary direction of PC1 (as is common practice), and to systematically eliminate problematic chromosomes where we could not be sure that PC1 is capturing compartmentalization. In more detail on the two points:*

*(1) Gene density is known to correlate with the active compartment “A” (PMID: 19815776). The direction of PCs are arbitrary (i.e. positive vs negative). Thus, it is standard practice to use gene density to orient the direction of PC1 such that positive values reflect compartment A (the relatively gene dense compartment), while negative values reflect compartment B. This convention allows for the comparison of compartments across samples.*

*(2) A/B compartments are almost always reflected by the first PC (i.e. PC1). This PCA is performed on each chromosome separately. We and others have seen that occasionally for acrocentric chromosomes (chr13, 14, 15, 21, 22) and chromosomes with exceptionally large centromeres (chr1 and 9) the first PC reflects the chromosome arms (i.e. short vs long), rather than A vs B compartments. In these cases, the compartments are reflected by PC2 or PC3. Standard practice in the field starting from the very first Hi-C study (Lieberman-Aidan 2009) has been to manually examine the first 3 PCs for each chromosome and select the one that matches the characteristic plaid pattern of compartments which is visually obvious (as opposed to the chromosome arms or other patterns). However, we agree with the reviewer’s comment #6 that manual inspection is not acceptable in a systematic study like ours. Thus, in order to standardize the determination that PC1 reflects the compartment pattern, we measured the correlation of each of the first 3 PCs with gene density along the chromosome. The true compartment patterns will be highly correlated with gene density as discussed above. To be conservative, if any chromosome had a first PC (i.e. PC1) that was not the PC most strongly correlated with gene density, we discarded that chromosome in all individuals for the PC1 analysis. In practice, this eliminated 3 of 5 acrocentric chromosomes (chr14, 21 and 22), both chromosomes with exceptionally large centromeres (chr1 and 9), and chromosome 19. It is not clear why chromosome 19 came up in this analysis, but it has been shown previously to have peculiar morphology (PMID: 10366586).*

6. Line 838-839 Manual inspection as a QC step is unacceptably irreproducible. If there are QC analysis or inspection steps, they should be coded up as computer instructions. These can be developed from manually obtained insight on the discovery samples, but not on samples considered as replication/validation.

*This was an unfortunate use of wording on our part. We removed this language. Indeed, we agree with the reviewer. All calculations and filtering steps were done systematically according to a set of rules (as described in more detail above re comment 5). In the original submission we included the phrase about confirmation by manual inspection to assure folks in the 3D genome community who are aware of this issue and accustomed to performing manual inspection. We have now removed this sentence, because no changes, calculations, or decisions related to compartment phenotyping were made as a result of any manual inspections.*

7. Fig 1a 2a 3a: In the red LD triangle plots: color scale should be the same for all samples'

*We replotted triangle plots in Figure 1a, 2a, 3s, S1a, S5a, S6f, and S7a such that all individuals within a figure are plotted on the same color scale. We also note that the red heat maps are not showing LD as suggested by the reviewer. They show Hi-C contact frequencies. We added a more explicit label to Figure 1 to make this clear.*

8. Fig 1c: the right panel should be removed. Everybody is reading this electronically, and can zoom in 3x if they want to.

*We agree that electronic versions can be easily zoomed in and out. However, we added the sub-panel on the right of 1c because at first glance at standard resolution it is easy to miss the separation between the lines at the genomic distances most relevant to gene regulation (<10Mb). We actually considered removing the left subpanel because contacts at longer distances are known to be less reproducible and have low overall correlation between samples, but we included it because we suspected that some readers might be interested in the full distributions. From our perspective, it is common practice to use insets or subpanels to highlight important sections of plots that are hard to see at the standard resolution at first glance. Therefore, we elected to leave this sub-panel in.*

9. Fig 1d is misleading as the significance threshold is not Bonferroni-corrected for the multiple testing of N=50 bars. The corrected threshold should be added, at -log10(p)=3. I agree with the authors that even at the relaxed threshold of nominal significance, -log10{p) = 1.3, as shown, there is enrichment of significant bars at each of the four 2.5Mb windows shown, but the bar-chart is still misleading to think each of the yellow bars is significant.

*We re-plotted with Bonferroni-corrected p value threshold.*

10. Fig1b: The Wilcoxon score shortchanges the significance. For INS and DI, the box-and-whiskers suggest that the top quartiles of the 20 within coefficients are higher than all of the 760 between coefficients. The p-value just for that is 20*19*18*17*16/(780*779*778*777*776) < 10^-13 .

*We thank the reviewer for pointing this out. We spotted an error in our code. We have now plot with re-calculated p-values from two-sided Wilcoxon rank sum test in Figure 1. We also now include both Wilcoxon rank sum tests and Kolmogorov–Smirnov tests in Supplemental Figure 4b-e.*

11. Limma is an expression data analysis package. It is not clear why this is the tool of choice to analyze 3D genome structure data. While statistics are statistics, no matter what, Limma incorporates normalizations, assumptions and distributions that are specific to RNAseq data, so one would need reassurances these are not unduly changing the results.

*The reviewer correctly points out that limma was initially designed for microarray gene expression data, and then extended to RNA-seq data. As a software package, limma contains different components including methods for normalization of microarrays. We were not clear in our original wording in the manuscript, but we did not use any of the normalization components of limma. The limma component we are using in our work is general code for fitting many standard linear models quickly. This component in not specific to microarrays or gene expression data. We also use an additional extension in limma, namely an Empirical Bayes variance shrinkage method (“eBayes”) which is well understood to improve results by borrowing strength across features (genes or in our case genomic bins), and is especially advantageous for small sample studies like ours. The end result is a statistical method very similar to ANOVA but with increased power for small number of replicates. To ensure that this approach is in general agreement with a standard ANOVA, we compared the F-statistics from limma to those from ANOVA (please see plots below). The Pearson correlation between limma and ANOVA is >0.93 in all cases. We have altered the text to clarify our use of limma in this context (lines 157-158)*

*We also note that limma or similar eBayes frameworks have been applied to data other than gene expression such as quantitative proteomics (PMID: 19087345), DNA methylation arrays (PMID: 25245051) and comparative ChIP-seq (PMID: 24852250).*

12. Fig 2: the presentation of a new set of 4 individuals is cherrypicking, which is an attempt to mislead. If the claims are correct, the authors should be able to demonstrate them with any four samples. They should stick to YRI-1,2,3 and 4.

*We assure that reviewer that there was no attempt anywhere in this manuscript to cherry-pick or mislead readers. We respectfully disagree that the example referred to here is cherry-picking or misleading. At this particular variable region, there is a strong upstream contact bias in the center of the region that is present to varying degrees in different individuals. We are clear in the text that in order to validate this region by FISH we chose two individuals who have a strong upstream contact bias at the variable DI region (YRI-8 and YRI-4), and two individuals who have a weak upstream contact bias at the variable DI region (YRI-5 and YRI-3). It would not have made sense to try to validate this variable region by choosing four individuals who have similar 3D structure at this region. We would not necessarily expect that any four individuals would be variable.*

*To address the reviewer’s concern, we have added contact maps and DI tracks for all 20 individuals at this region to Supplemental Figure 6a-b so readers can see the full range of variation at this region.*

13. Power analysis for detecting effects at p=0.05 is irrelevant as this is not the analysis that actually takes place

*We have included power analyses at the p-values corresponding to the FDR 0.2 cutoff for each QTL search.*

14. L799 ration -> ratio

*We thank the reviewer for the careful read. We have corrected this typo.*

**Reviewer #3:**

This is a very nice study, carefully done and expertly analyzed. I reviewed the statistical analysis carefully, and was pleased to note the attention to details, including batch and confounder correction, distinction between correlation and cause, and proper hypothesis driven tests. My comments are truly minor, with the exception of the first one:

1. Because of the small sample size, I am troubled by the confounding effects of population and potential other correlates on the QTL analysis. I suggest to remove the data from the samples that are not Yoruban individuals and confirm that the QTL analysis is robust. One way to test for this is to check that pairs of individuals do not share QTL genotypes more often than expected by chance (namely, that there is no unexpected structure in the QTL data).

*We thank the reviewer for recognizing the care taken in our study and feedback on how to improve it. We appreciate the reviewer's concern about potential confounding effects of population on the QTL analysis. Although we did use all 20 LCLs in in our initial “variation” analysis in Figures 1-3 (intended to characterize and give us an upper bound on the variation in the 3D genome irrespective of genetic influence), the QTL searches were performed exclusively on 11 unrelated YRI individuals for exact reason suggested by the reviewer. The non-YRI individuals were not included in the QTL “discovery”. They were used only in the “validation” set.*

*To further address the reviewer’s concern, we performed the suggested analysis by looking at whether QTL genotypes are shared more often than non-QTL genotypes (to the best of our understanding). More specifically: for a given SNP, we performed pairwise comparisons between all 11 YRI individuals in our QTL discovery set, and calculated the fraction of pairwise comparisons that share the same genotype (we refer to this metric as “shared genotype fraction” below). In the plots below, we show the shared genotype fraction as a function of minor allele frequency (MAF) in our discovery set. We do not observe significant differences in shared genotype fraction between QTL SNPs and non-QTL SNPs (i.e. tested but not identified as QTLs). p-values below are calculated by two-sided Wilcoxon rank sum test, though we note that the p-values are only approximate due to the number of ties present in the data.*

Much more minor comments:

1. It is not clear to me how they used limma to perform F tests (comparisons of variation within and between individuals).

*We recognize the need to clarify our use of limma. One component of limma is efficient code for fitting many F tests simultaneously by taking advantage of the fact that the design matrix is constant across bins. We also use an empirical Bayes variance shrinkage estimator, which makes our approach similar – but not identical – to F-tests in fixed effect models. The empirical Bayes estimator is well understood to increase power in small sample studies like ours, by borrowing information across bins. Please see our response to reviewer 2 comment 11 for additional details on this point.*

2. I wonder how robust are the QTLs you identified to other approaches to characterize the Hi-C data (for example, if you analyzed the contact frequencies by using another normalization method).

*We agree this is an interesting question. To address this, we renormalized the Hi-C contact matrices for all individuals using KR matrix balancing (which is a commonly used alternative to HiCNorm), and then re-evaluated the genotype-phenotype relationship at our QTLs. We performed this analysis at 3 different resolutions (i.e bin sizes; 25Kb, 50Kb, and 100Kb) to address the reviewer’s next point as well. In Supplemental Figure 9c we compare the genotype-phenotype slope from our original QTL search (i.e. the linear mixed effects model (LMM)) to the slope of a linear genotype-phenotype regression model (LM)*

*using the recalculated contact matrices. In every comparison, we find that the LMM slopes from our QTL search and the LM slope using the recalculated data are strongly and significantly correlated, suggesting that these QTLs are robust to Hi-C normalization method and resolution.*

3. Similarly, I wonder about the robustness of the results with respect to different data resolution.

*We agree this is also an interesting question. Please see our response to point #2 above, and Supplemental Figure 9c.*

4. It was not clear to me how replicates were considered in the QTL analysis.

*We apologize for a lack of clarity. We have added better description to the methods along with a link to the code. Briefly, in the QTL analysis, we fitted a linear mixed effects regression model, treating the 3D chromatin phenotypes (i.e., FIRE score, DI score, IS score, chromatin interaction frequency) as the outcome variable, and the genotype as the explanatory variable. In the model fitting, we used 11 unrelated YRI individuals, with two biological replicates for each individual. Since two biological replicates from the same individual are dependent, we used an individual-specific random effect to specifically characterize such within-individual dependence. We used the “gls” function in the R package “nlme” to fit the linear mixed effects model. Using the replicates in this way enhances the power of our QTL search, relative to simply pooling the replicates for each individual and then analyzing 11 independent observations derived from the pooled samples. Our R code is available at http://renlab.sdsc.edu/renlab_website//download/iqtl/.*
